# Impact of Iron Supplementation on Clinical Outcomes in Iron-Deficient Heart Failure Patients: A Retrospective Cohort Study in Oman

**DOI:** 10.3390/jcm15166142

**Published:** 2026-08-07

**Authors:** Dania Abunaser, Juhaina S. Al Maqbali, Salim Al Busaidi, Yousra Nomier, Yousuf Al Suleimani

**Affiliations:** 1 Department of Pharmacology and Clinical Pharmacy, College of Medicine & Health Sciences, Sultan Qaboos University, Muscat 123, Oman; y.nomeir@squ.edu.om; 2 Department of Pharmacy, Sultan Qaboos University Hospital, University Medical City, Muscat 123, Oman; juhaina@squ.edu.om; 3 Department of Medicine, Sultan Qaboos University Hospital, University Medical City, Muscat 123, Oman; salimb@squ.edu.om

**Keywords:** iron deficiency, heart failure, intravenous-iron, ferric carboxymaltose, readmission, mortality

## Abstract

**Objective**: The prevalence and clinical impact of iron deficiency (ID) in patients with heart failure (HF) in Oman remain insufficiently characterized. This study aimed to evaluate the prevalence of ID and its association with clinical outcomes, as well as to assess the impact of iron supplementation on hospitalization and mortality among HF patients. **Methods**: A retrospective observational cohort study included adult patients (≥18 years) with HF who had ≥2 iron profiles; patients with end-stage renal disease were excluded. ID was defined using American Heart Association (AHA) and European Society of Cardiology (ESC) criteria. **Results**: Of 2246 patients screened, 211 met inclusion criteria (57.8% female; mean age 70 ± 12.7 years). ID prevalence was 68.3% (AHA) and 71.1% (ESC), with ESC criteria detecting more biochemical deficiencies. ID was associated with higher all-cause and HF-related readmissions (*p* < 0.01), longer length of stay (*p* = 0.045), and more atrial fibrillation (AF) (*p* < 0.01). All-cause mortality was similar, although the composite of mortality and readmission was slightly higher with ID (*p* = 0.052). Iron supplementation significantly increased ferritin (+32 ng/mL) and transferrin saturation (+2.8%) (*p* < 0.01) and was linked to fewer strokes (*p* = 0.049), with no major differences in other outcomes. **Conclusions**: The study found that ID was highly prevalent; however, systematic screening was not routinely practiced, which largely led to delayed initiation of appropriate management. Routine ESC-guided ID screening and management are recommended. However, given the retrospective, non-randomized study design, these findings should not be interpreted as evidence of a causal treatment effect and are likely influenced by confounding by indication. Prospective randomized studies are needed to confirm these observations.

## 1. Introduction

Heart failure (HF) remains a leading cause of morbidity and mortality worldwide [1]. In addition to standard neurohormonal therapies, growing attention has focused on managing comorbidities that contribute to disease progression. One such condition is iron deficiency (ID), which affects nearly 50% of HF patients and is linked to reduced exercise tolerance, poorer functional status, and higher hospitalization rates [2]. Over the past decade, multiple randomized trials have evaluated the benefits of intravenous (IV) iron therapy in HF, particularly ferric carboxymaltose (FCM). Studies such as FAIR-HF, CONFIRM-HF, and AFFIRM-AHF proved improvements in exercise capacity and symptoms, and in some cases reduced HF hospitalizations, though mortality benefits remain unproven [3,4,5]. Although the large HEART-FID trial did not meet its composite primary goal of death or cardiovascular hospitalization, it did confirm beneficial impacts on iron indices [6]. Consequently, current European Society of Cardiology (ESC) (2021) and the American Heart Association (AHA) (2022) guidelines recommend IV iron in symptomatic HF with reduced ejection fraction (HFrEF) and ID to enhance functional status, while acknowledging that effects on survival are unknown [7,8]. Oral iron is widely used to treat ID in the general population, yet in HF it is often ineffective due to impaired absorption from gut edema, inflammation, and elevated hepcidin. The IRONOUT-HF trial found that oral iron did not lead to significant improvements, highlighting that IV iron remains the more effective treatment option [9]. However, oral iron is still commonly used in situations where IV treatment is either too expensive or not easily accessible. In the Middle East, the use of IV iron is often limited by factors such as restricted infusion facilities, low awareness, and cost concerns. In Oman, where anemia and hereditary blood disorders are relatively common, treatment often relies on oral iron supplements or blood transfusions instead. Oman provides universal access to healthcare through a predominantly government-funded healthcare system. Despite substantial improvements in public health indicators, anemia and iron deficiency remain prevalent because of a combination of nutritional, chronic disease-related, and demographic factors. Patients with heart failure are generally managed according to international guideline recommendations; however, routine screening for and treatment of iron deficiency may vary across healthcare settings. This variability highlights the importance of evaluating current clinical practice and outcomes in the Omani population. However, this study aimed to assess how iron supplementation affects clinical outcomes in Omani HF patients with ID. We hypothesized that iron supplementation, particularly IV iron, would be associated with reduced hospitalizations and potentially lower mortality compared with no supplementation. The study aimed to generate real-world evidence that complements international trial findings and supports improved clinical practice in the region.

## 2. Materials and Methods

### 2.1. Study Population

**Figure 1:**
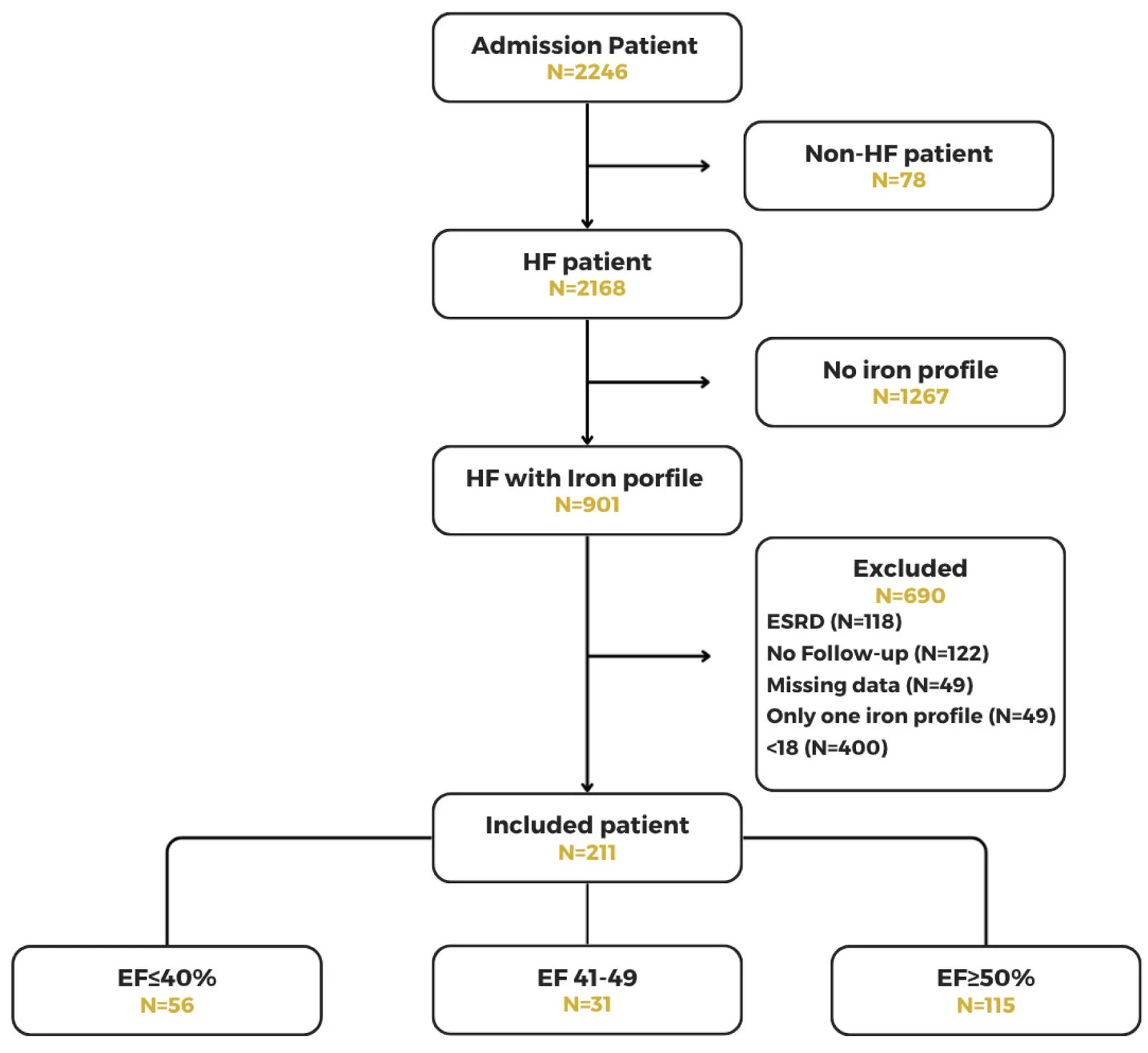
Flowchart of patient selection from the hospital database between January 2020 and January 2023. A total of 2246 patients with heart failure were screened, of whom 211 met the eligibility criteria and were included in the final analysis.

This analysis included HF patients identified as ID from the retrospective cohort study. Patients with at least two iron profiles between 2020 and 2023 were studied. Eligible participants included adults aged 18 years or older with a confirmed diagnosis of HF who had a complete iron profile assessment at both baseline and follow-up to enable longitudinal analysis. Patients were excluded if they were younger than 18 years, had incomplete medical records (such as missing baseline iron data or echocardiography findings), had only a single hospital visit without follow-up, or had end-stage renal disease (ESRD) or were undergoing dialysis. The final cohort was selected following careful screening of all hospital records to ensure compliance with the inclusion and exclusion criteria Figure 1. Patients were followed from the date of the baseline iron profile until the last available clinical follow-up, death, or the end of the study period (January 2023), whichever occurred first.

### 2.2. Demographic Variables

Age, biological sex, and nationality were extracted from the electronic medical record where available. The study population included only Omani nationals. Sex was analyzed as a biological variable. Race and ethnicity were not routinely recorded in the medical record and were unavailable for analysis. Age eligibility was restricted to adults aged 18 years or older, with patients younger than 18 years excluded.

### 2.3. Data Collection and Definitions

The patients were categorized into three main groups: (1) HF patients with untreated ID, (2) those with treated ID, and (3) those without ID. All patients met the criteria for ID at baseline, based on either the ESC or AHA definitions, which showed a strong level of agreement between them. In addition, patients were stratified into two subgroups based on the administration of iron supplementation during hospitalization (treated vs. untreated). Data extraction captured comprehensive demographic, clinical, laboratory, and therapeutic information. Demographic and comorbidity data included age, sex, and the presence of hypertension, diabetes mellitus, chronic kidney disease (CKD), and cardiovascular disease. Laboratory variables included hemoglobin, serum ferritin, serum iron, transferrin saturation (TSAT), transferrin, C-reactive protein (CRP), serum creatinine, estimated glomerular filtration rate (eGFR), and N-terminal pro-B-type natriuretic peptide (NT-proBNP). Laboratory measurements were obtained from the hospital’s accredited clinical laboratory using standardized analytical methods. Complete blood count parameters, including hemoglobin, were measured using automated flow cytometry. Serum ferritin was determined using an electrochemiluminescence sandwich immunoassay, while transferrin concentrations were measured by an immunoturbidimetric assay. Serum iron concentrations were determined using a colorimetric FerroZine-based method following release of iron from transferrin under acidic conditions. TSAT was calculated using the formula: TSAT (%) = 3.982 × serum iron/transferrin. CRP was measured as part of the routine biochemical assessment and was included among the laboratory variables analyzed. Left ventricular ejection fraction (LVEF) was assessed using transthoracic echocardiography performed on a Philips CVx ultrasound system according to standard institutional protocols. Quantitative assessment of LVEF was performed using transthoracic echocardiography during admission. LVEF was classified as preserved (≥50%), mid-range (40–49%), or reduced (<40%) and was assessed at both baseline and follow-up. Functional status was classified according to the New York Heart Association (NYHA) functional classification where available. Medication use, including angiotensin-converting enzyme (ACE) inhibitors, angiotensin receptor blockers (ARBs), angiotensin receptor–neprilysin inhibitors (ARNIs), beta-blockers, mineralocorticoid receptor antagonists (MRAs), sodium-glucose cotransporter-2 (SGLT2) inhibitors (primarily dapagliflozin), anticoagulants, antiplatelets, statins, and loop diuretics, was also documented. Patients received guideline-directed medical therapy for HF according to contemporary clinical practice and at the discretion of the treating physicians. Detailed data on iron therapy were recorded, specifying the formulation, dose, route, and duration of treatment.

### 2.4. Definitions

Key operational definitions were established to maintain consistency across analyses. ID was defined based on both the AHA-HF and ESC-HF guidelines. According to the AHA-HF, ID was defined as a serum ferritin level below 100 μg/L or a ferritin level between 100 and 299 μg/L combined with TSAT below 20% [7], whereas in the ESC-HF, ID was defined as either ferritin below 100 μg/L or ferritin below 400 μg/L with TSAT under 20% [10]. When ferritin data were unavailable, ID was defined as serum iron below 10 μg/dL and TSAT below 20%, or as clinically documented in the TrackCare system. These definitions were applied retrospectively for research classification and comparative analytical purposes using the most contemporary criteria available at the time of data analysis; they were not used to guide treatment decisions during the original period of patient care To improve readability and avoid duplication, analyses based on the ESC definition were selected for presentation in the main manuscript, while corresponding analyses based on the AHA definition were included in the Supplementary Materials as sensitivity analyses assessing the consistency of findings across definitions. Anemia was defined according to World Health Organization (WHO) criteria as hemoglobin below 13 g/dL in men and below 12 g/dL in women. Iron supplementation was classified into two categories: oral iron (primarily ferrous sulfate) and IV iron (including ferric carboxymaltose (FCM), iron sucrose, or iron saccharate) depending on clinical indication and local availability. The effectiveness of supplementation was evaluated through changes in iron indices, hemoglobin levels, and clinical outcomes over time. Iron supplementation was prescribed at the discretion of the treating physician as part of routine clinical care and was not assigned according to a standardized study protocol. Treatment decisions were based on the patient’s clinical condition, laboratory evidence of ID and/or anemia, and physician judgment. Clinical outcomes included hospitalization frequency, cause-specific mortality, and incidence of cardiovascular events. The number of emergency visits, hospital readmissions, and deaths after discharge and throughout follow-up were recorded.

### 2.5. Statistical Analysis

A comprehensive statistical analysis was performed to evaluate the associations between ID, iron supplementation, and clinical outcomes among patients with HF. Descriptive statistics were used to summarize baseline characteristics. Continuous variables were expressed as means and standard deviations (SD) for normally distributed data, or as medians with interquartile ranges (IQR) for skewed distributions, as determined by the Shapiro–Wilk test. Categorical variables were presented as frequencies and percentages. For group comparisons, one-way analysis of variance (ANOVA) was used to compare normally distributed continuous variables across the three study groups, whereas the Kruskal–Wallis test was applied for non-normally distributed variables. The Chi-square test or Fisher’s exact test was used to evaluate differences in categorical variables. For two-group comparisons, independent *t*-tests were used for normally distributed variables, while the Mann–Whitney U test was used for non-normally distributed variables. Absolute changes in key clinical parameters such as EF, hemoglobin, ferritin, TSAT, and creatinine from baseline to follow-up were analyzed using ANOVA or Kruskal–Wallis tests, with paired comparisons evaluated through *t*-tests or Mann–Whitney U tests as appropriate. 

Multivariable binary logistic regression models were constructed to identify independent predictors of hospital admission, emergency department visits, composite adverse outcomes, mortality, and ID. Candidate variables included those associated with the respective outcome at *p* < 0.05 in univariable analyses, together with clinically relevant covariates selected a priori based on the literature, including age, sex, body mass index, diabetes mellitus (DM), hypertension (HTN), anemia, and HF subtype. Backward stepwise elimination was used to derive the final models, with a significance level of *p* < 0.05 applied for variable retention. Clinically important confounders were retained in the models regardless of their statistical significance. Before model fitting, multicollinearity among candidate predictors was evaluated using variance inflation factors and tolerance statistics. A variance inflation factor greater than 5 or a tolerance value below 0.20 was considered indicative of potentially problematic multicollinearity. No substantial multicollinearity was identified among the variables included in the final models. The results of the multivariable logistic regression analyses are reported as adjusted odds ratios, with corresponding 95% confidence intervals and *p*-values. To reduce selection bias, confounding, and baseline imbalances between treatment groups, propensity score matching was performed. Propensity scores were estimated using multivariable logistic regression based on clinically relevant baseline covariates, including age, sex, body mass index, heart failure subtype, history of transient ischemic attack or stroke, anemia status, ferritin level, transferrin saturation, and other relevant baseline characteristics. Patients were matched in a 1:1 ratio using nearest-neighbor matching without replacement and a caliper width of 0.2 standard deviations of the logit of the propensity score. Covariate balance before and after matching was evaluated using standardized mean differences and variance ratios. Adequate balance was defined as an absolute standardized mean difference below 0.10 for each matching variable. Love plots and summary balance statistics were used to assess covariate balance visually and quantitatively and to confirm comparability between the matched groups. Kaplan–Meier survival curves were generated to compare mortality outcomes, with statistical differences assessed using the log-rank test. Furthermore, Cox proportional hazards regression models were used to evaluate the associations of ID and iron supplementation with survival outcomes. The results are reported as hazard ratios with corresponding 95% confidence intervals. No statistically significant violations of the proportional hazards assumption were detected. All statistical analyses were conducted using STATA version 14.2 (StataCorp, College Station, TX, USA), and a two-sided *p*-value < 0.05 was considered significant.

## 3. Results

### 3.1. Prevalence and Baseline Characteristics 

Over a median follow-up of 1.0 year (IQR 0.5–1.8), ID was highly prevalent, affecting 68.3% of patients according to AHA criteria and 71.1% according to ESC criteria. Anemia was present in 93.4% of the cohort, with 66.4% exhibiting combined ID and anemia, while isolated ID without anemia was uncommon (4.3%). Baseline demographic and clinical features of the cohort were similar across ID status groups overall (*p* > 0.05). However, a higher proportion of women was observed among ID patients compared to non-ID (*p* < 0.05). Treated ID patients were more likely to have heart failure with preserved ejection fraction (HFpEF) and demonstrated higher mean LVEF compared with untreated ID and non-ID groups (*p* < 0.01 under both definitions). Obesity and hypertension were more prevalent among treated ID patients, and hydralazine use was significantly higher in this group (*p* < 0.01). Conversely, prior stroke or TIA was more common in patients without ID (*p* < 0.01). NYHA functional class was available for a subset of patients and did not differ significantly between the study groups. Overall, patients who received iron supplementation had a greater burden of comorbidities and more severe disease characteristics at baseline than untreated patients, suggesting that treatment allocation reflected routine clinical practice rather than random assignment. Detailed baseline characteristics are presented in Table 1. The corresponding analyses based on the AHA definition are presented in Supplementary Table S1.

### 3.2. Clinical Outcomes by Iron Deficiency Status 

During follow-up, patients with ID experienced more adverse events, particularly if untreated. Compared with patients without ID, both treated and untreated ID groups had higher rates of emergency department visits, HF-related emergency visits, HF-related hospitalizations, and all-cause hospital admissions, as seen in Table 2. Untreated ID patients generally experienced the highest HF-related event rates, whereas treated ID patients demonstrated intermediate rates. All-cause hospital admissions were common among patients with ID. Those who received iron treatment experienced the highest admission rates (96.7%), followed by untreated ID patients (90.5%) and non-ID individuals (77.3%) (*p* < 0.01). Mortality was comparable between untreated ID and non-ID patients (25.4% and 26.1) but slightly higher among treated ID patients (31.7%). The composite adverse outcome, comprising death, hospitalization, or emergency visits, was also most frequent in the treated ID group (98.4%) versus the non-ID group (87.8%, *p* = 0.028 by ESC criteria). Atrial fibrillation (AF) was more prevalent among treated ID patients (~35%) compared with the other groups (*p* < 0.01). Iron-treated patients showed significant improvements in ferritin, serum iron, and TSAT; however, these biochemical improvements did not completely offset the higher observed rates of adverse clinical outcomes, as seen in Table S3. Detailed outcome comparisons are summarized in Table 2.

### 3.3. Clinical Outcomes by Iron Supplementation 

Among the 211 study participants, 101 (47.9%) received iron supplementation, and 110 (52.1%) did not. Clinical outcomes differed by treatment status. Patients who received iron had a higher all-cause hospital admission rate than those who did not (92.1% vs. 81.8%, *p* = 0.028) and a longer median hospital stay (*p* = 0.004). There was a trend toward fewer HF-related hospitalizations in the supplemented group (56.4% vs. 59.1%), but this difference was not significant. Interestingly, the risk of cardiovascular death (including HF-specific mortality) was significantly lower in patients who did not receive iron (10.0% vs. 19.8% in supplemented, *p* = 0.045). Composite adverse events (death, HF hospitalization, or emergency visit) occurred in almost all patients but were more frequent among those given iron (98.0% vs. 89.1% without iron, *p* = 0.011). Similarly, AF incidence was significantly higher in the supplemented group (27.7% vs. 10.9%, *p* < 0.01), whereas stroke incidence was lower with iron therapy (5.0% vs. 12.7%, *p* = 0.049), as seen in Table 3. Details of biochemical changes can be seen in Table S4.

**Figure 2:**
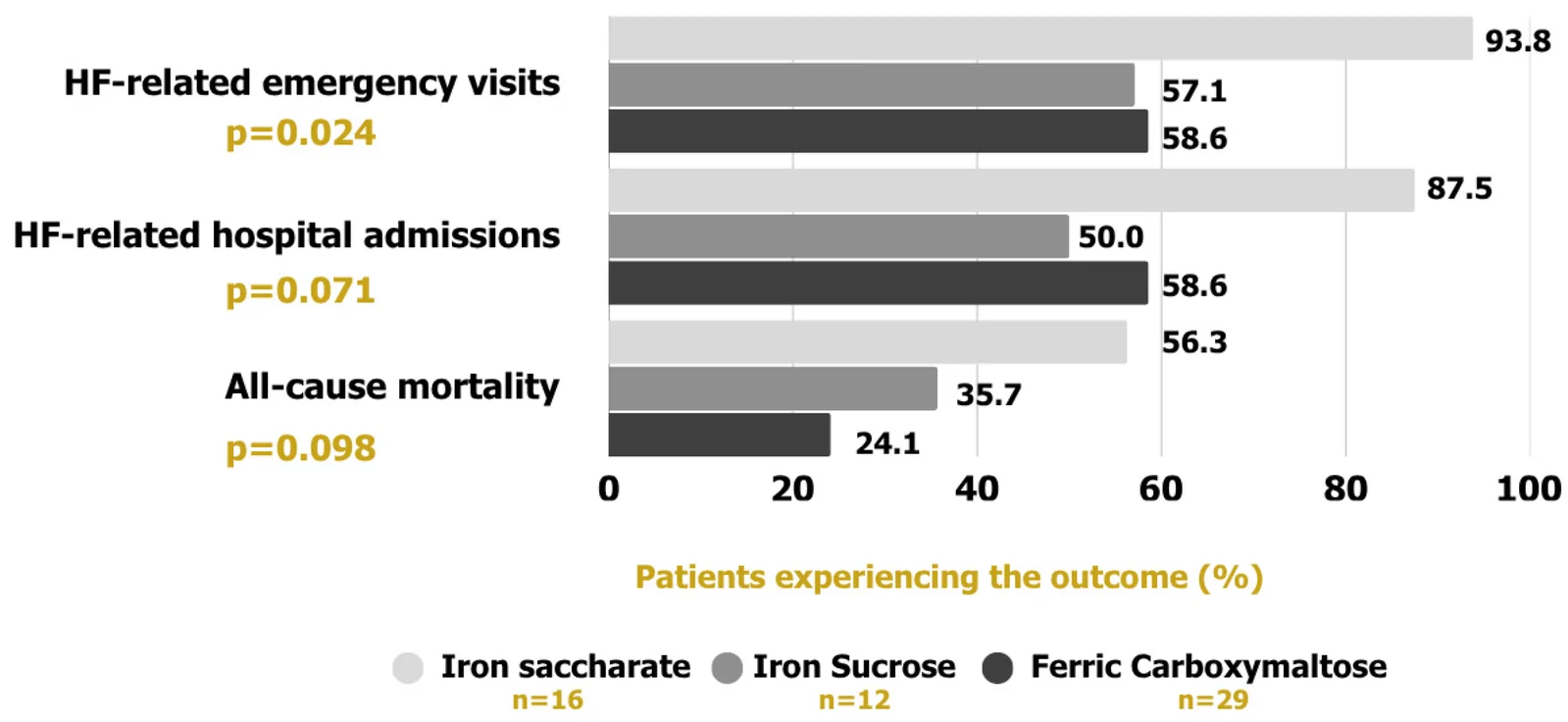
Clinical outcomes among patients receiving different intravenous iron formulations.

Patients who received IV iron had higher observed event rates than those who received oral iron. For example, patients treated with IV iron had the highest composite event rate (100%) compared to oral supplementation (95.3%) and no supplementation (89.1%, *p* = 0.022). IV-treated patients also had more frequent hospitalizations (96.6% vs. 85.7% with oral, *p* = 0.025) and longer stays (median 3 days vs. 2 days, *p* < 0.01). Cardiovascular emergency visits and AF occurred at substantially higher rates in the IV group (CVD emergencies in 33.9% IV vs. 7.1% oral, *p* < 0.01; AF in 35.6% IV vs. 16.7% oral, *p* < 0.01). Consistent with the ID group analysis, iron supplementation (particularly IV) led to greater improvements in ferritin and transferrin saturation levels, yet these biochemical gains did not clearly translate into better clinical outcomes. An additional analysis focusing on IV iron formulations revealed that emergency visits and hospital admissions were common across all types, affecting more than 95% of patients. Interestingly, HF-related emergencies were markedly higher among those treated with saccharate (94%) compared with patients receiving iron sucrose or FCM (around 58%, *p* = 0.024). Although total admissions and mortality were slightly higher in the saccharate group, these differences did not reach statistical significance. However, patients treated with FCM tended to have lower mortality rates, fewer cardiovascular deaths, and fewer HF-related emergency visits. Cardiovascular events, including AF (36%) and myocardial infarction (12%), occurred at similar rates across all IV. An additional analysis examined different intravenous (IV) iron formulations. Figure 2 shows that emergency department visits and hospital admissions were frequent across all types, occurring in more than 95% of patients. Notably, HF-related emergency visits were significantly higher among patients treated with iron saccharate (94%) compared with those receiving iron sucrose or ferric carboxymaltose (FCM) (approximately 58%, *p* = 0.024). Although overall hospital admissions and mortality rates were slightly higher in the saccharate group, these differences were not statistically significant. In contrast, patients treated with FCM demonstrated a trend toward lower mortality and fewer HF-related emergency visits Table S5.

### 3.4. Survival Analysis

Kaplan–Meier survival analysis by iron status (untreated ID, treated ID, vs. non-ID) showed no statistically significant differences in survival between groups. This finding was consistent across both the AHA and ESC definitions of ID Figures S1 and S2. One-year survival rates were similar regardless of whether patients had ID or received treatment (*p* > 0.05). In contrast, survival differed by iron supplementation status. Patients who received iron therapy had a significantly lower survival rate over time compared to those who did not receive supplementation. The Kaplan–Meier curve highlights this difference, showing that patients who received iron supplementation had a 1.71-fold higher risk of death compared to those who did not (HR = 1.71, 95% CI 1.01–2.88, *p* = 0.045), as seen in Figure S3. When stratified by type of supplementation (IV iron, oral iron, or none), the survival curves show that non-supplemented patients had the highest survival rate, IV-supplemented patients had the lowest survival rate, and oral-supplemented patients had an intermediate survival rate. A Cox proportional hazards model confirmed that oral supplementation was associated with a significantly better survival compared to IV supplementation (HR = 0.734 vs. IV, 95% CI 0.55–0.98, *p* = 0.036), as seen in Figure S4.

### 3.5. Subgroup Analysis by Heart Failure Phenotype

#### 3.5.1. Heart Failure with Reduced Ejection Fraction

Clinical outcomes and biochemical markers among patients with heart failure with reduced ejection fraction (HFrEF) were evaluated by dividing them into three groups, untreated ID, treated ID, and non-ID, based on both AHA and ESC definitions. Emergency visits were common across all groups, with untreated ID patients showing the highest rates (AHA: 66.7%; ESC: 63.2%), though the differences were not statistically significant. Similarly, HF-related hospitalizations were most frequent in untreated ID patients (AHA: 72.2%; ESC: 73.7%). The treated ID group, which included only seven patients, had the lowest rate of cardiovascular-related admissions (26.7%), but this was not statistically significant. Mortality was comparable among all groups. Other cardiovascular events such as stroke, TIA, and AF were infrequent and mostly absent among treated patients Table S6. Patients with heart failure with mid-range ejection fraction (HFmrEF) were not analyzed separately because of the relatively small sample size (n = 31), which limited meaningful subgroup comparisons.

#### 3.5.2. Heart Failure with Preserved Ejection Fraction

Among patients with preserved EF, ID was significantly linked to worse cardiovascular outcomes. Patients with HFpEF who were ID experienced more cardiovascular-related hospital admissions compared to those without ID. For example, under AHA criteria, 46.2% of ID HFpEF patients (especially those treated with iron) had a CVD hospitalization, versus only 20.9% in non-ID HFpEF (*p* = 0.042). A similar significant difference was observed using ESC criteria (*p* = 0.035). AF was also much more common in HFpEF patients with ID; the ID patients (particularly those who received iron) had AF in 43% of cases compared to 11% in non-ID HFpEF (*p* < 0.01), as seen in Tables S8 and S9. Although patients treated with IV iron for HF were more likely to experience emergency admissions, HF-related hospitalizations, and AF than those who received oral supplementation. 

Figure 2 shows the proportions of patients experiencing HF-related emergency department visits, HF-related hospital admissions, and all-cause mortality among those treated with ferric carboxymaltose (n = 29), iron sucrose (n = 12), and iron saccharate (n = 16). *p*-values represent comparisons across the three treatment groups. Because of the relatively small sample sizes within each treatment subgroup, these analyses are exploratory and should be interpreted with caution.

### 3.6. Predictors of Adverse Outcomes and Iron Deficiency

#### 3.6.1. Predictors of Clinical Outcomes

**Figure 3:**
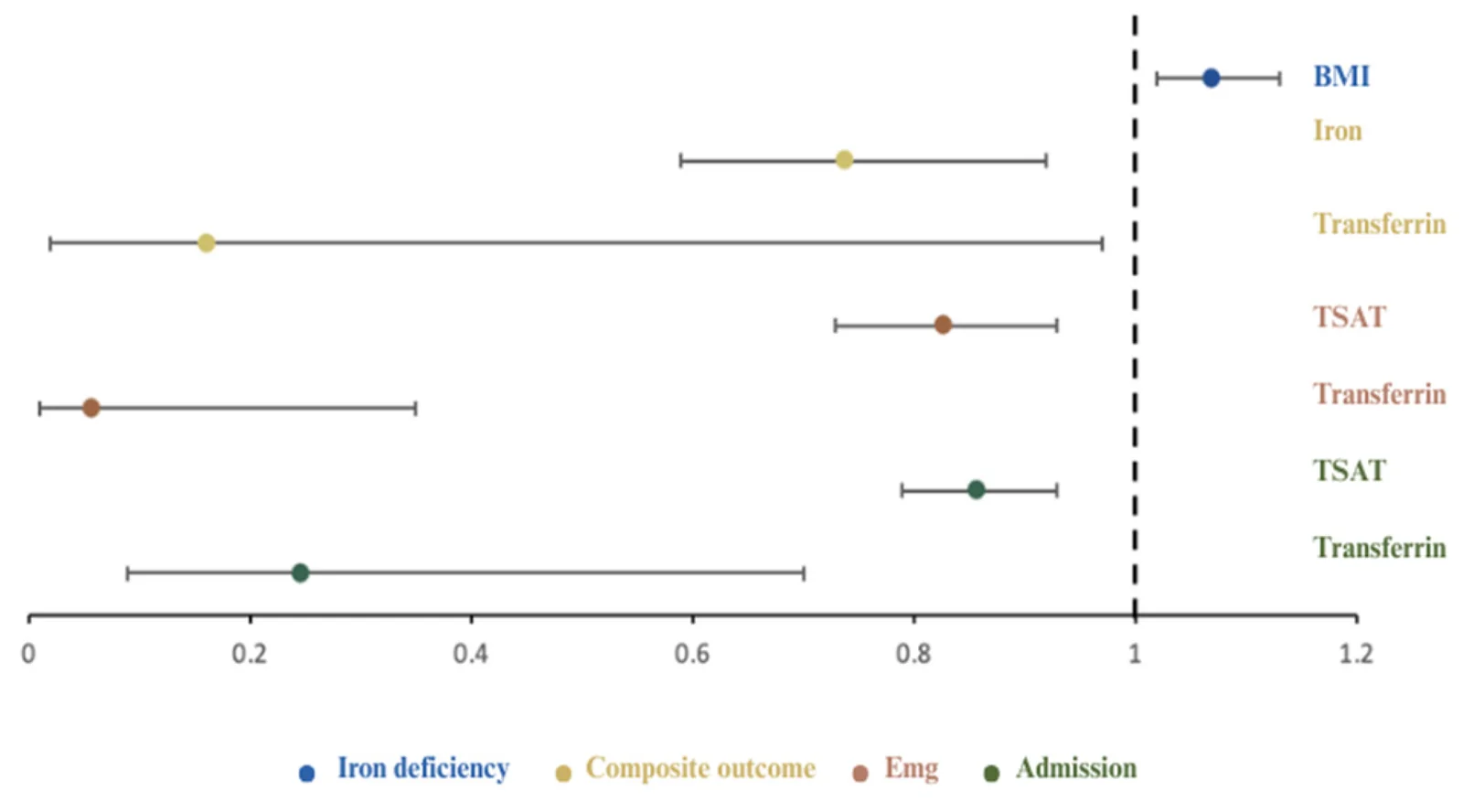
Associations between iron parameters and clinical outcomes in heart failure patients. The vertical dashed line indicates the null value (odds ratio = 1), representing no association between the iron parameter and the clinical outcome.

Multivariable logistic regression showed baseline iron markers independently predicted adverse HF outcomes, as seen in Table S10. Lower transferrin was associated with higher hospitalization (OR = 0.246, 95% CI 0.09–0.70, *p* < 0.01) and emergency visit risk (OR = 0.057 for reduced/normal vs. elevated, *p* < 0.01). Lower TSAT also predicted hospital admissions (OR = 0.858 per %, *p* < 0.01) and emergency visits (OR = 0.827, *p* < 0.01). For composite events, low transferrin was protective (OR 0.161, *p* = 0.046), while low serum iron increased risk (OR ~1/0.738 ≈ 1.35, *p* < 0.01), as seen in Figure 3. Propensity score matching (PSM) addressed confounding; 60 treated patients were matched to 47 controls (n = 107), improving baseline balance. In the matched cohort, no significant differences were observed in hospitalizations (90.0% vs. 85.1%, *p* = 0.442), emergency visits (95.0% vs. 95.7%, *p* = 0.856), or composite events (96.7% vs. 95.7%, *p* = 0.803). Before entropy balancing, substantial imbalance was observed in several baseline covariates. After restriction to the common support and entropy balancing for the average treatment effect on the treated, covariate balance improved markedly. Absolute standardized mean differences were below 0.10 for all covariates except hemoglobin (SMD = 0.118), indicating good overall balance with minor residual imbalance in hemoglobin Table S11.

#### 3.6.2. Predictors of Iron Deficiency

Independent predictors of having ID were also explored. Among baseline variables, higher BMI emerged as a significant predictor of ID status in HF patients (OR = 1.07 per 1 kg/m^2^ increase, 95% CI 1.02–1.13, *p* < 0.01). No other strong independent predictors of ID were reported in Figure 3.

A forest plot analysis showing adjusted ORs and 95% confidence intervals CIs from the multivariable logistic regression analyses identifying independent predictors of hospital admission, emergency department visits, composite adverse outcomes, and ID. Odds ratios below 1 indicate a lower likelihood of the outcome, whereas values above 1 indicate a higher likelihood.

## 4. Discussion

This study provides the first national evaluation of ID among patients with HF in Oman, demonstrating its high prevalence and significant association with adverse clinical outcomes. Patients treated with FCM showed improvements in iron indices and fewer HF-related hospitalizations; however, no clear survival benefit was observed, possibly because of the limited sample size and follow-up duration. The use of both AHA and ESC definitions showed that the findings were generally consistent across contemporary diagnostic criteria, supporting the robustness of the observed associations between ID and clinical outcomes. These analyses were intended to assess the consistency of the findings across diagnostic definitions rather than to compare guideline performance and should therefore be considered exploratory.

In summary, our findings highlight a substantial burden of ID and anemia among Omani patients with HF. Despite this high prevalence, iron status was infrequently assessed, with routine screening performed in less than half of the patients, indicating that many cases of ID remained unrecognized until anemia became apparent. Among those who were identified and treated with iron supplementation (primarily IV iron), iron stores were successfully replenished, and there was a modest trend toward fewer HF-related emergency visits. However, we did not observe clear improvements in long-term outcomes, as treated patients had no evident survival benefit and even higher rates of adverse clinical events, likely because therapy was often initiated in older, more advanced cases. Finally, in evaluating risk factors, a higher BMI emerged as an independent predictor of ID, suggesting that overweight and obese HF patients are particularly prone to developing ID in this population.

This nationwide study found a strikingly high rate of ID and anemia among Omani patients with HF. Using both AHA and ESC criteria, roughly two-thirds to three-quarters of patients had ID, similar to international reports, but at the higher end of what’s usually reported [11,12]. Even more notable, anemia affected over 90% of patients, far higher than what most global studies describe [13,14,15]. This high prevalence of anemia should be interpreted within the Omani context, where population-specific and healthcare-related factors may contribute to its burden. Hereditary hemoglobin disorders, including sickle cell trait and β-thalassemia trait, are relatively common in Oman and may lead to chronic anemia or microcytosis. Nutritional factors, such as variable dietary iron intake and possible micronutrient deficiencies, may further increase anemia risk [16]. In addition, HF care in Oman is often hospital-based, and systematic screening for ID is not consistently integrated into routine practice. Together, these factors may delay the recognition of ID until anemia becomes clinically evident, partly explaining the high anemia prevalence observed in this cohort [17,18]. Even though ID was common, fewer than half of patients (41.6%) had their iron status assessed, and testing was often deferred until anemia had already developed. Notably, this percentage includes patients with only a single iron profile documented, underscoring how rarely routine, systematic screening occurred. This points to a clear gap between guideline recommendations and everyday clinical practice. Both AHA and ESC criteria define ID independently of the hemoglobin level; an anemia-first testing strategy likely caused many cases of ID to be missed or identified late. Such delays may be particularly concerning in settings where hemoglobinopathies are prevalent, since baseline microcytosis or chronic anemia can obscure coexisting ID and complicate interpretation of laboratory results [19,20]. Early detection, regardless of hemoglobin levels, could improve treatment timing, maximize therapeutic efficacy, and enhance clinical outcomes. It is worth noting that our screening rate (41.6%) was higher than international reports (e.g., <25% in the Swedish HF Registry, 2018) and prior studies [19,20], underscoring the need for standardized protocols and greater clinical awareness to improve early detection and management of ID in HF. Patients receiving iron therapy were typically older, more often female, and more likely to have HFpEF, consistent with evidence linking ID to HFpEF through inflammation-related hepcidin elevation and impaired oxygen utilization [11,21,22]. In this cohort, ID was linked to worse outcomes, especially more HF-related emergency visits (>70%) and hospital admissions (>90%) among untreated ID, supporting the idea that ID is an important risk marker in HF and contributes to symptoms, reduced physical capacity, and greater hospitalization risk even when anemia is not present [3,23]. Among those who received iron therapy, iron markers improved meaningfully (ferritin, transferrin saturation, and serum iron rose), showing that iron stores were successfully replenished. However, iron-treated patients also had higher hospitalization and mortality rates than untreated or non-ID patients. While this differs from clinical trials (where IV iron has reduced HF hospitalizations) [3,4,5], the most likely explanation is confounding by indication in real-world settings; clinicians tend to treat older, sicker patients with more advanced HF and heavier comorbidity burdens. Because iron supplementation was prescribed as part of routine clinical practice rather than through randomized treatment allocation, patients who received iron were more likely to have clinical indications such as ID, anemia, or greater disease severity. These baseline differences may have contributed to their poorer outcomes independently of the treatment itself. Iron therapy was also frequently initiated during hospitalization rather than at an earlier stage, suggesting delayed recognition of ID. In these patients, the severity of the underlying illness may have outweighed any short-term clinical benefit of iron replacement. Healthcare system-related gaps may have also contributed to the high hospitalization and ED visit rates. The absence of a structured HF clinic and consistent early follow-up may have led patients to return to the ED for relatively mild symptoms, regardless of iron status, thereby potentially obscuring any benefit of iron therapy in this cohort. Therefore, the observed associations should not be interpreted as evidence of harm from IV iron therapy but rather as findings that may be explained by confounding by indication and residual baseline imbalances.

Iron therapy was associated with a modest, non-significant reduction in HF-related emergency visits but higher mortality and adverse events, likely reflecting greater baseline disease severity rather than harm from treatment. These results differ from clinical trials showing clear benefits of IV iron [24], probably due to real-world factors such as early adoption, delayed initiation, suboptimal dosing, and inconsistent follow-up. During the study period, adherence to guideline-directed ID management in Oman was inconsistent, mirroring global practice gaps. Despite these limitations, the observed trend suggests potential benefit and underscores the importance of treating ID in HF patients regardless of anemia, in line with current guidelines. Iron-treated patients had a significantly lower stroke incidence than untreated patients (5.0% vs. 12.7%, *p* = 0.049), suggesting a potential protective effect mediated by improved endothelial function, mitochondrial metabolism, oxygen utilization, and greater anticoagulant use [25,26]. In contrast, AF was more frequent among iron-treated patients (27.7% vs. 10.9%, *p* < 0.01), likely reflecting more advanced HF, anemia, and HFpEF rather than an adverse treatment effect. Prior evidence links anemia and ID to AF through impaired myocardial energetics and atrial remodeling [27,28], indicating that underlying disease severity is the primary driver and highlighting the need for further prospective studies. Patterns by iron formulation also suggest changing practice over time. Although differences were not statistically significant overall, outcomes tended to favor FCM compared with older preparations like iron sucrose or saccharate, which matches the stronger trial evidence and practical advantages of FCM (including simpler dosing and a reassuring safety profile) [3,4,24]. Mortality patterns in our cohort also tended to favor FCM, though differences were not statistically significant (*p* = 0.098). Cardiovascular deaths followed a similar trend. These findings align with FCM’s well-established safety record [29,30]. Worse outcomes were observed with saccharate, which is consistent with previous studies [31]. This likely reflects earlier use in more severely ill patients rather than lower effectiveness. Iron sucrose and saccharate were used earlier in the study period, while FCM was introduced later and showed improved hospitalization and functional outcomes, consistent with randomized trials. Patient selection remains a potential confounder. Furthermore, comparisons among different iron formulations should be interpreted cautiously because the relatively small number of patients in each treatment subgroup limited the statistical power to detect meaningful differences. These analyses should therefore be regarded as exploratory.

Subgroup analyses suggested that the response to iron therapy may vary according to HF phenotype. In patients with HFrEF, iron treatment appeared to be associated with reduced HF readmissions, in line with previous trial evidence [24]. Importantly, iron supplementation in patients with HFpEF was primarily prescribed for the treatment of documented iron deficiency and/or anemia rather than as guideline-directed therapy for HFpEF itself. Current HF guidelines do not support the routine administration of iron solely for the management of HFpEF. Therefore, the poorer outcomes observed among patients with HFpEF who received iron may reflect greater baseline disease severity, a higher comorbidity burden, and confounding by indication rather than a harmful effect of iron therapy. Given the relatively small number of patients within each HF phenotype subgroup, the study was not adequately powered to detect differential treatment effects. Accordingly, these subgroup findings should be considered exploratory and hypothesis-generating and interpreted cautiously until confirmed in larger prospective studies.

BMI was also an independent predictor of ID, with each 1 kg/m^2^ increase associated with higher odds of ID (OR = 1.07, 95% CI 1.02–1.13, *p* < 0.01). Although obesity is often associated with higher dietary iron intake, this finding supports the obesity iron paradox, whereby chronic inflammation increases hepcidin and reduces iron absorption and availability [32,33]. Thus, overweight and obese HF patients may represent a high-risk group requiring careful iron monitoring.

Several limitations should be acknowledged. First, the retrospective design is inherently subject to selection bias and confounding. Despite multivariable adjustment and propensity score matching (PSM), residual confounding cannot be excluded, as unmeasured or incompletely measured variables may have influenced both treatment allocation and clinical outcomes. The relatively small sample size also limited the statistical power of the subgroup and survival analyses, while the follow-up period may have been insufficient to detect longer-term treatment benefits. In addition, only 211 of the 2246 patients initially screened met the inclusion criteria because complete iron-profile measurements were required at both baseline and follow-up. Consequently, the study cohort may represent a selected subgroup of patients who underwent more intensive clinical evaluation or follow-up and may not be fully representative of the broader heart failure population. This selection process may have introduced selection bias and limited the generalizability of the findings. Treatment timing, dosing, and follow-up assessments were not standardized, which may have obscured potential clinical effects. Furthermore, treatment allocation was based on physician judgment rather than randomization, introducing the possibility of confounding by indication. Patients who received iron supplementation were generally older, had poorer iron indices, a greater burden of comorbidities, and more advanced disease. These baseline differences may have influenced the observed clinical outcomes independently of iron therapy. Overall, iron deficiency appears to be highly prevalent yet underrecognized among Omani patients with heart failure and is associated with greater healthcare utilization. Although iron therapy appears effective in restoring iron stores, delayed and selective treatment—often initiated in patients with more advanced disease—may limit its real-world clinical impact. More consistent guideline-based screening and earlier intervention may improve outcomes and reduce the burden of heart failure in this high-risk population.

## 5. Conclusions

ID is highly prevalent among patients with HF in Oman and is associated with increased hospitalizations, emergency department visits, and adverse clinical outcomes. Despite its clinical importance, routine screening for ID remains suboptimal, potentially resulting in delayed diagnosis and management. Although iron supplementation, particularly IV iron, improved biochemical markers of ID, its observed association with poorer clinical outcomes should be interpreted cautiously. Given the retrospective observational design and the likelihood of confounding by indication, these findings should not be considered evidence of harm from iron therapy. Rather, they may reflect the greater baseline disease severity and comorbidity burden among patients selected for treatment. These findings highlight the importance of early, guideline-directed screening and timely management of ID in patients with HF. The subgroup findings in patients with HFpEF and other HF phenotypes should be considered exploratory and require confirmation in adequately powered prospective studies. Future research should prioritize prospective multicenter cohorts or randomized controlled trials evaluating iron supplementation in patients with HF and ID. Standardized treatment protocols, longer follow-up periods, and adequately powered subgroup analyses, particularly in HFpEF populations, are needed to validate these findings, reduce the effects of residual confounding, and better identify the patients most likely to benefit from iron therapy.

## Figures and Tables

**Table 1:** Baseline characteristics of HF study patients regardless of HF type by ESC-HF ID definition.

Category	ESC-HF ID Definition				
	Overall	Untreated ID	Treated ID	Not ID	*p*
	n = 211	n = 65	n = 85	n = 61	
Duration of follow-up, IQR, year (^‡^)	1 (0.5–1.8)	1.1 (0.6–2.2)	1 (0.5–1.5)	1.11 (0.5–2.5)	**0.222**
**Patient history (n, %)**					
Age (years) (^†^)	69.9 ± 12.7	69.2 ± 12.4	72.2 ± 12.1	67.4 ± 13.5	0.069
Female	122 (57.8)	42 (64.6)	52 (61.2)	28 (45.9)	0.075
**History of cardiovascular disease (n, %)**					
IHD	53 (25.1)	13 (20.0)	22 (25.9)	18 (29.5)	0.459
PVD	2 (1.00)	0 (0.00)	1 (1.2)	1 (1.6)	0.751
AF (fibrillation)	58 (27.5)	17 (26.2)	27 (31.8)	14 (23.0)	0.480
CAD	42 (19.9)	12 (18.5)	17 (20.0)	13 (21.3)	0.923
HTN	173 (82.0)	54 (83.1)	75 (88.2)	44 (71.1)	**0.043**
MI	23 (10.9)	6 (9.2)	9 (11.0)	8 (13.1)	0.778
**History (n, %)**					
CKD, renal failure	123 (58.3)	32 (49.2)	56 (65.9)	35 (57.4)	0.121
Dyslipidemia	52 (24.6)	18 (27.7)	23 (27.1)	11 (18.0)	0.363
DM	141 (66.8)	44 (67.7)	60 (70.6)	37 (60.7)	0.447
BMI (^‡^)	28.9 (24.1–31.2)	28.8 (25.1–32.1)	28.9 (25.1–32.2)	27.2 (22.8–28.9)	**0.015**
Stroke/TIA	21 (10.0)	8 (12.3)	2 (2.4)	11 (18.0)	**<0.01**
Anemia	197 (93.4)	58 (89.2)	83 (97.7)	56 (91.8)	0.085
**Type of HF (n, %)**					
HFrEF	65 (30.8)	21 (32.3)	20 (23.5)	24 (39.3)	0.118
HFmidEF	31 (14.7)	10 (15.4)	7 (8.2)	14 (23.0)	**0.046**
HFpEF	115 (54.5)	34 (52.3)	58 (68.2)	23 (37.7)	**<0.01**
**Clinical characteristics (n, %) FC (NYHA)**					
I	1 (3.9)	1 (9.1)	0 (0.00)	0 (0.0)	1.000
II	14 (53.9)	5 (45.5)	4 (50.0)	5 (71.4)	0.612
III	8 (30.8)	4 (36.4)	3 (37.5)	1 (14.3)	0.648
IV	3 (11.5)	1 (9.1)	1 (12.5)	1 (14.3)	1.000
**Cause of HF (n, %)**					
Ischemic	23 (10.9)	9 (13.9)	7 (8.2)	7 (11.5)	0.543
Dilated cardiomyopathy	13 (6.2)	4 (6.2)	4 (4.7)	5 (8.2)	0.673
Unknown	175 (82.9)	52 (80.0)	74 (87.1)	49 (80.3)	0.425
**Echocardiography (n, %)**					
EF (%) (^‡^)	50 (35–60)	50 (35–60)	55 (40–60)	45 (30–55)	**0.049**
LVEF (%) (^†^)	48.4 ± 14.1	47.5 ± 13.9	52.1 ± 14.1	44.2 ± 14.1	**<0.01**
**Supplementation (n, %)**					
Iron therapy	101 (47.9)	0 (0.00)	85 (100)	16 (26.2)	**<0.01**
**Baseline Analytical and Pharmacological Data**					
eGFR (mL/min/1.73 m^2^) (^‡^)	53 (37–81)	60 (41–84)	48 (33–67)	62 (40–77)	0.064
Creatinine (mg/dL) (^‡^)	100 (72–125)	91 (66–126)	114 (75–144)	97 (75–125)	0.186
CRP (mg/L) (^‡^)	25 (6–76)	28.5 (6.5–71)	14.5 (4–70)	32 (6–79)	0.508
NT-proBNP (pg/mL) (^‡^)	2574 (1182–6263)	1936.5 (876.5–6603.5)	3226 (1320–8737)	2918.5 (1583–4963)	0.533
SBP (mmHg) (^†^)	132.4 ± 24.3	132.7 ± 23.5	134.1 ± 23.8	129.6 ± 26.2	0.532
DBP (mmHg) (^†^)	68.5 ± 13.1	67.6 ± 13.0	69.3 ± 13.2	68.1 ± 13.3	0.723
Hemoglobin (g/dL) (^†^)	9.5 ± 1.8	9.9 ± 1.8	8.9 ± 1.6	9.8 ± 2.0	**<0.01**
Ferritin (ug/L) (^‡^)	123.5 (42–282)	78 (30.6–152)	78 (26–163)	434 (251.5–740)	**<0.01**
Iron (umol/L) (^‡^)	6 (4–9)	6 (5–8)	4 (3–7)	10 (8–14)	**<0.01**
Transferrin (g/L) (^†^)	2.2 ± 0.7	2.3 ± 0.7	2.4 ± 0.6	1.7 ± 0.6	**<0.01**
TSAT (%) (^‡^)	12.1 (7.4–19.9)	11.1 (8.1–16.1)	7.8 (5.2–12.6)	23.4 (20.1–30.4)	**<0.01**

Data are expressed as % of cases and mean ± SD for normally distributed variables (^†^) and median (IQR) for abnormally distributed variables (^‡^). *p*-values < 0.05 were considered statistically significant (bold). Comparisons among the three study groups (untreated ID, treated ID, and non-ID) were performed using one-way ANOVA or the Kruskal–Wallis test for continuous variables and the Chi-square or Fisher’s exact test for categorical variables, as appropriate; ESC-HF, European Society of Cardiology; IHD, ischemic heart disease; PVD, peripheral vascular disease; AF, atrial fibrillation; AF, atrial flutter, CAD, coronary artery disease HT, hypertension; MI, myocardial infarction; CKD, chronic kidney disease; DM, diabetes mellitus; COPD, chronic obstructive pulmonary disease; BMI, body mass index; HFrEF, heart failure with reduced ejection; HFmrEF, heart failure with mid-range ejection fraction; HFpEF, heart failure with preserved ejection; and NYHA, New York Heart Association (n = 26), and it is presented for descriptive purposes only. It was not included in comparative analyses because of the high proportion of missing data. HF, heart failure; EF, ejection fraction; LVEF, left ventricular ejection fraction; eGFR, estimated glomerular filtration rate; CRP, C-reactive protein (n = 153); SBP, systolic blood pressure; DBP, diastolic blood pressure; Ferritin (ng/mL) (n = 136); and TSAT, transferrin saturation level test (n = 201). Detailed baseline medication use is presented in Supplementary Table S2.

**Table 2:** Clinical adverse outcomes and biochemical changes across ID groups.

Category	ESC-HF ID Definition				
	Total Number	Untreated ID	Treated ID	Not ID	*p*-Value
	n = 211	n = 66	n = 63	n = 82	
**Clinical adverse outcome**					
**Emergency visits (n, %)**					
Total Emg	193 (91.5)	63 (95.5)	61 (96.8)	69 (84.2)	**0.010**
Total number of Emg (^‡^)	3 (1–6)	3 (2–6)	3 (2–7)	3 (1–5)	0.164
HF-Emg	126 (59.7)	47 (71.2)	40 (63.5)	39 (47.6)	**0.011**
CVD-Emg	46 (21.8)	15 (22.7)	16 (25.4)	15 (18.3)	0.576
**Admission (n, %)**					
Total admission	183 (86.7)	59 (89.4)	61 (96.8)	63 (76.8)	**<0.01**
Length of hospital stay (days) (^‡^)	2 (1–4)	2 (1–3)	3 (2–4)	2 (1–4)	**0.045**
HF admission	122 (57.8)	45 (68.2)	38 (60.3)	39 (47.6)	**0.037**
CVD admission	65 (30.8)	18 (27.3)	26 (41.3)	21 (25.6)	0.097
**HF decompensation (n, %)**					
Arrhythmia	2 (1.00)	1 (1.5)	1 (1.6)	0 (0.0)	0.526
Infection	65 (30.8)	22 (33.3)	20 (31.8)	23 (28.1)	0.772
Ischemic	0 (0.0)	0 (0.0)	0 (0.0)	0 (0.0)	-
Unknown	64 (30.3)	24 (36.4)	18 (28.6)	22 (26.8)	0.426
All-cause mortality (n, %)	58 (27.5)	16 (24.2)	21 (33.3)	21 (25.6)	0.455
**Reason of death (n, %)**					
HF	19 (9.0)	7 (10.6)	6 (9.5)	6 (7.3)	0.774
CVD	12 (5.7)	1 (1.5)	6 (9.5)	5 (6.1)	0.125
Other	26 (12.3)	7 (10.6)	9 (14.3)	10 (12.2)	0.816
Composite outcome (n, %)	197 (93.4)	63 (95.5)	62 (98.4)	72(87.8)	**0.028**
**CVD occurrence (n, %)**					
IHD	2 (1.0)	1 (1.5)	0 (0.0)	1 (1.2)	1.000
Stroke	19 (9.0)	8 (12.1)	5 (7.9)	6 (7.3)	0.561
MI	19 (9.0)	8 (12.1)	5 (7.9)	6 (7.3)	0.561
TIA	7 (3.3)	4 (6.1)	2 (3.2)	1 (1.2)	0.303
AF	40 (19.0)	6 (9.1)	22 (34.9)	12 (14.6)	**<0.01**

Data are presented as percentages of cases and median (IQR) for abnormally distributed variables (^‡^). *p*-values < 0.05 were considered statistically significant (bold). Comparisons among the three study groups (untreated ID, treated ID, and non-ID) were performed using one-way ANOVA or the Kruskal–Wallis test for continuous variables and the Chi-square or Fisher’s exact test for categorical variables, as appropriate, regardless of HF classification or patient follow-up conditions; ESC-HF, European Society of Cardiology; Emg, emergency visit; CVD, cardiovascular vascular disease; HF, heart failure; IHD, ischemic heart disease; MI, myocardial infarction; TIA, transient ischemic attack; AF, atrial fibrillation.

**Table 3:** Impact of iron supplementation on clinical adverse outcomes and biochemical parameters.

Category	Total Number n = 211	Supplemented n = 101	Not Supplemented n = 110	*p*-Value
**Duration of iron supplementation, IQR, years (^‡^)**	0 (0–12)	12 (43–22)	0 (0–0)	**<0.01**
**Clinical adverse outcome**				
**Emergency visits (n, %)**				
Total Emg	193 (91.5)	96 (95.1)	97 (88.2)	0.074
Total number of Emg (^‡^)	3 (1–6)	3 (2–6)	3 (1–6)	0.324
HF-Emg	126 (59.7)	61 (60.4)	65 (59.1)	0.847
CVD-Emg	46 (21.8)	23 (22.8)	23 (20.9)	0.743
**Admission (n, %)**				
Total admission	183 (86.7)	93 (92.1)	90 (81.8)	**0.028**
Length of hospital stay (days) (^‡^)	2 (1–4)	3 (2–4)	2 (1–3)	**0.004**
HF admission	122 (57.8)	57 (56.4)	65 (59.1)	0.696
CVD admission	65 (30.8)	36 (35.6)	29 (26.4)	0.145
**HF decompensation (n, %)**				
Arrhythmia	2 (1.00)	1 (1.00)	1 (0.9)	1.000
Infection	65 (30.8)	32 (31.7)	33 (30.0)	0.791
Unknown	64 (30.3)	30 (29.7)	34 (30.9)	0.849
**All-cause mortality (n, %)**	58 (27.5)	34 (33.6)	24 (21.8)	0.054
**Reason of death (n, %)**				
HF	19 (9.0)	11 (10.9)	8 (7.3)	0.359
CVD	12 (5.7)	9 (8.9)	3 (2.7)	0.073
Cardiovascular death (HF and CVD combined)	31 (17.7)	20 (19.8)	11 (10.0)	**0.045**
Other	26 (12.3)	14 (13.9)	12 (10.9)	0.515
**Composite outcome (n, %)**	197 (93.4)	99 (98.0)	98 (89.1)	**0.011**
**CVD occurrence (n, %)**				
IHD	2 (1.00)	1 (1.00)	1 (0.9)	1.000
Stroke	19 (9.00)	5 (5.0)	14 (12.7)	**0.049**
MI	19 (9.00)	10 (9.9)	9 (8.2)	0.663
TIA	7 (3.3)	2 (2.00)	5 (4.6)	0.448
AF	40 (19.00)	28 (27.7)	12 (10.9)	**<0.01**

Data are expressed as percentages of cases. Each patient was exclusively assigned to one group. Variables are presented as percentages, and median with interquartile range (IQR) for non-normally distributed data (^‡^). A *p*-value < 0.05 was considered statistically significant (bold). *p*-values compare patients who received iron supplementation with those who did not. Continuous variables were analyzed using independent-samples *t*-tests or Mann–Whitney U tests, and categorical variables were analyzed using chi-square or Fisher’s exact tests, as appropriate. (Analysis includes all HF types). Emg, emergency visit; CVD, cardiovascular disease; HF, heart failure; IHD, ischemic heart disease; MI, myocardial infarction; TIA, transient ischemic attack; AF, atrial fibrillation.

## Data Availability

Data are available from the corresponding authors upon reasonable request and institutional approval.

## Supplementary Materials

The following supporting information can be downloaded at https://www.mdpi.com/article/10.3390/jcm15166142/s1, Figure S1: Kaplan–Meier survival curves comparing overall survival according to iron deficiency status defined using the AHA-HF criteria (untreated iron deficiency, treated iron deficiency, and no iron deficiency). Figure S2: Kaplan–Meier survival curves comparing overall survival according to iron deficiency status defined using the ESC-HF criteria (untreated iron deficiency, treated iron deficiency, and no iron deficiency). Figure S3: Kaplan–Meier survival curves showing overall survival according to iron supplementation status (supplemented vs. not supplemented) in patients with heart failure. Figure S4: Kaplan–Meier survival curves showing overall survival among patients with heart failure according to iron supplementation type (intravenous iron, oral iron, and no iron supplementation). Figure S5: Kaplan–Meier survival curves comparing overall survival according to type of iron supplementation in patients with heart failure. Figure S6: Love plot showing covariate balance before and after entropy balancing. Table S1: Baseline demographic, clinical, laboratory, and treatment characteristics of the study population according to the AHA-defined iron deficiency status in patients with heart failure. Table S2: Selected clinical history, device therapy, and medication characteristics of the study population according to the ESC-defined iron deficiency status in patients with heart failure. Table S3: Changes in biochemical, echocardiographic, and clinical parameters according to AHA- and ESC-defined iron deficiency status in patients with heart failure. Table S4: Changes in echocardiographic, biochemical, and clinical parameters according to iron supplementation status in patients with heart failure. Table S5: Clinical outcomes according to intravenous iron formulation among iron-supplemented patients with heart failure. Table S6: Comparison of Clinical Adverse Outcomes Between Iron Deficiency (ID), Treated Iron Deficiency (Treated ID), and Non-Iron Deficiency (Non-ID) Groups in Patients with Heart Failure with Reduced Ejection Fraction (HFrEF). Table S7: Comparison of Clinical Adverse Outcomes Between Iron Deficiency (ID) and Non-Iron Deficiency (Non-ID) Groups in Patients with Heart Failure with Reduced Ejection Fraction (HFrEF). Table S8: Comparison of Clinical Adverse Outcomes Between Iron Deficiency (ID), Treated Iron Deficiency (Treated ID), and Non-Iron Deficiency (Non-ID) Groups in Patients with Heart Failure with Preserved Ejection Fraction (HFpEF). Table S9: Comparison of Clinical Adverse Outcomes Between Iron Deficiency (ID) and Non-Iron Deficiency (Non-ID) Groups in Patients with Heart Failure with Preserved Ejection Fraction (HFpEF). Table S10: Multivariable logistic regression analysis for predictors of iron deficiency, hospital admission, emergency department visits, and composite outcome. Table S11: Covariate balance before and after entropy balancing.

## Abbreviations

The following abbreviations are used in this manuscript:

ACEI	Angiotensin-Converting Enzyme Inhibitors
AF	Atrial Fibrillation
AHA	American Heart Association
ANOVA	Analysis of Variance
ARB	Angiotensin Receptor Blockers
ARNI	Angiotensin Receptor–Neprilysin Inhibitors
BB	Beta Blockers
BMI	Body Mass Index
CAD	Coronary Artery Disease
CCB	Calcium Channel Blockers
CI	Confidence Interval
CKD	Chronic Kidney Disease
COPD	Chronic Obstructive Pulmonary Disease
CRP	C-Reactive Protein
CVD	Cardiovascular Disease
DBP	Diastolic Blood Pressure
DM	Diabetes Mellitus
EF	Ejection Fraction
eGFR	Estimated Glomerular Filtration Rate
Emg	Emergency Visit
ESC	European Society of Cardiology
ESRD	End-Stage Renal Disease
FCM	Ferric Carboxymaltose
HF	Heart Failure
HFpEF	Heart Failure with Preserved Ejection Fraction
HFmrEF	Heart Failure with Mid-Range Ejection Fraction
HFrEF	Heart Failure with Reduced Ejection Fraction
HR	Hazard Ratio
HTN	Hypertension
ICD	Implantable Cardioverter-Defibrillator
ID	Iron Deficiency
IHD	Ischemic Heart Disease
IQR	Interquartile Range
IV	Intravenous
LVEF	Left Ventricular Ejection Fraction
MI	Myocardial Infarction
MRA	Mineralocorticoid Receptor Antagonists
NT-proBNP	N-terminal pro-B-type Natriuretic Peptide
NYHA	New York Heart Association
OR	Odds Ratio
PPI	Proton Pump Inhibitors
PSM	Propensity Score Matching
PVD	Peripheral Vascular Disease
SBP	Systolic Blood Pressure
SD	Standard Deviation
SGLT2	Sodium-Glucose Co-Transporter 2
TIA	Transient Ischemic Attack
TSAT	Transferrin Saturation
WHO	World Health Organization
