# Supplementary material for: Impact of Iron Supplementation on Clinical Outcomes in Iron-Deficient Heart Failure Patients: A Retrospective Cohort Study in Oman
Source: J Clin Med. 2026 Aug 7;15(16):6142. doi: 10.3390/jcm15166142 (PMC13513479; doi:10.3390/jcm15166142)
Supplement: Supplementary file 1 [file jcm-15-06142-s001.zip › jcm-4366642-supplementary.pdf]

**Supplementary Figure S1.** Kaplan–Meier survival curves comparing overall survival according to iron deficiency status defined using the AHA-HF criteria (untreated iron deficiency, treated iron deficiency, and no iron deficiency). The hazard ratio (HR), 95% confidence interval (CI), and log-rank p-value are shown.

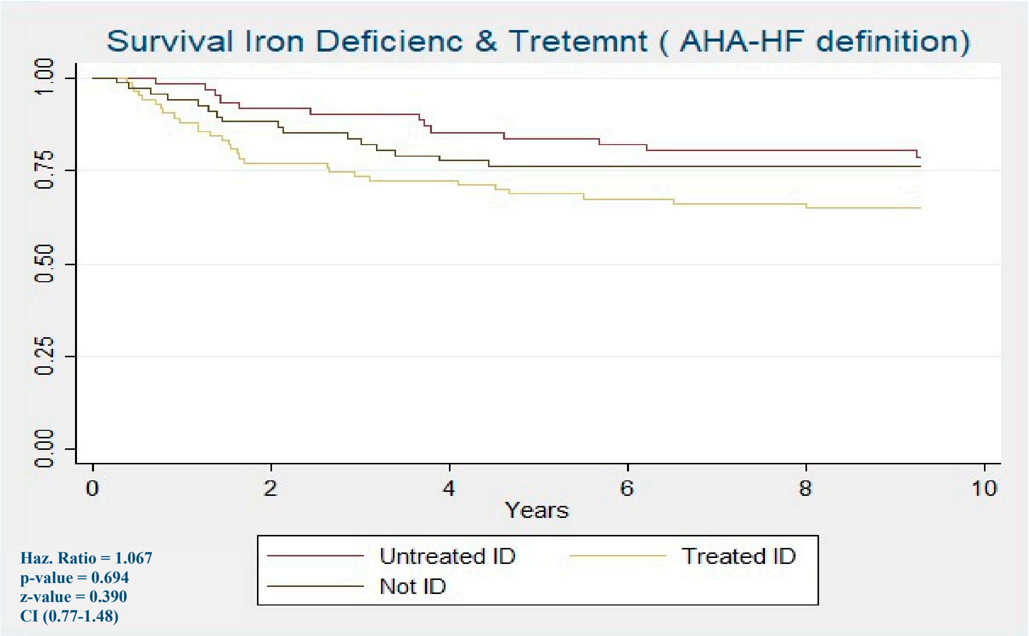


**Supplementary Figure S2**. Kaplan–Meier survival curves comparing overall survival according to iron deficiency status defined using the ESC-HF criteria (untreated iron deficiency, treated iron deficiency, and no iron deficiency). The hazard ratio (HR), 95% confidence interval (CI), and log-rank p-value are shown.

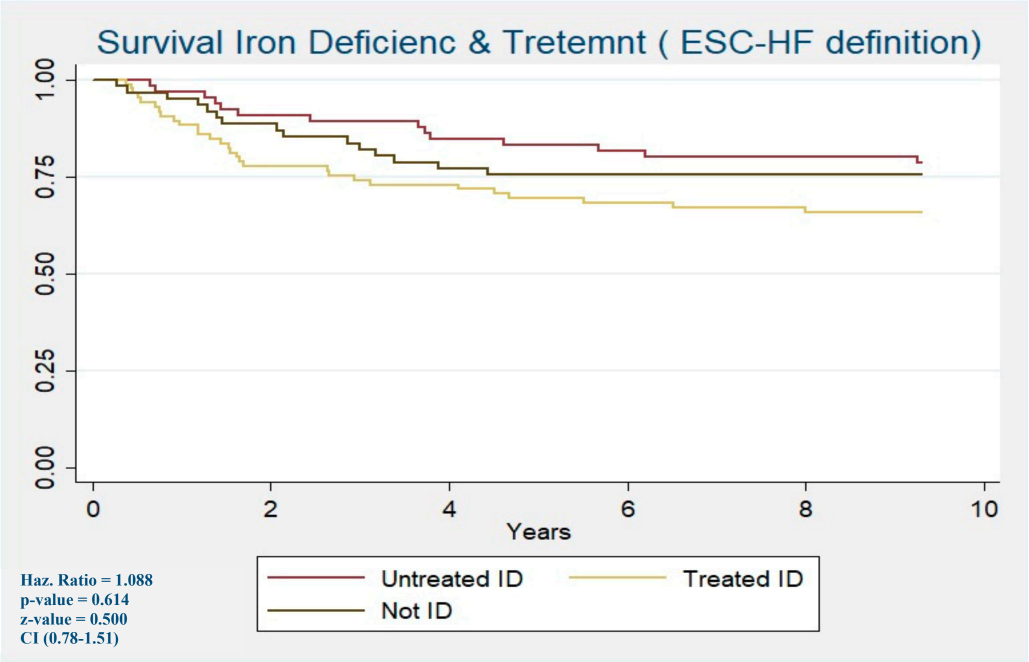

**Supplementary Figure S3.** Kaplan–Meier survival curves showing overall survival according to iron supplementation status (supplemented vs. not supplemented) in patients with heart failure. The x-axis represents follow-up time (years), and the y-axis represents cumulative overall survival probability. Hazard ratio (HR) = 0.734, 95% confidence interval (CI) 0.55–0.98; log-rank p = 0.036.

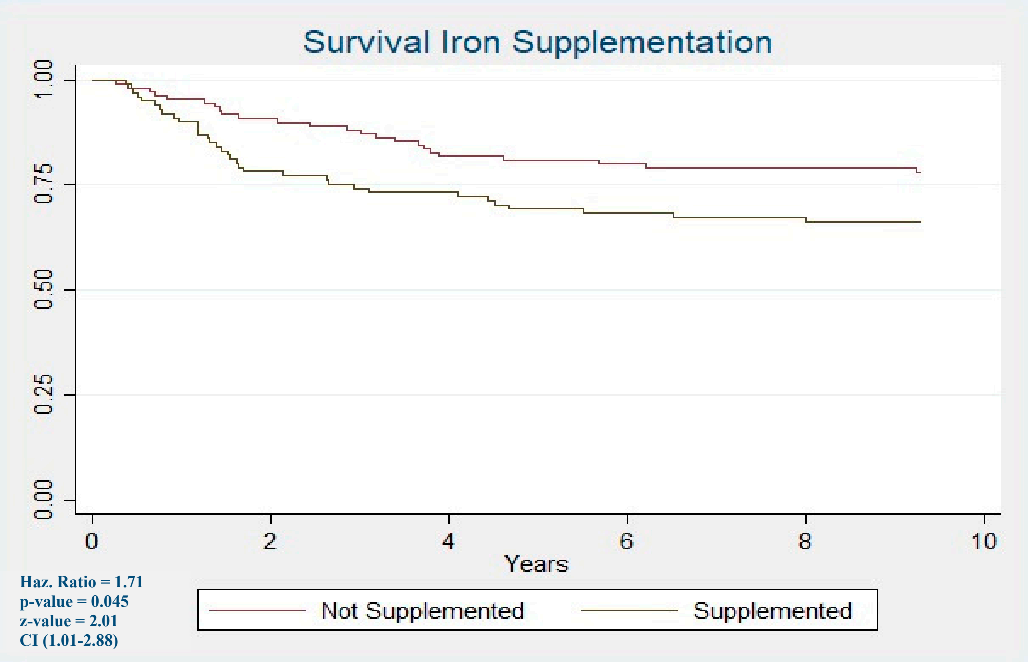


**Supplementary Figure S4.** Kaplan–Meier survival curves showing overall survival among patients with heart failure according to iron supplementation type (intravenous iron, oral iron, and no iron supplementation). The x-axis represents follow-up time (years), and the y-axis represents cumulative overall survival probability. Hazard ratio (HR) = 0.734, 95% confidence interval (CI) 0.55–0.98; log-rank p = 0.036.

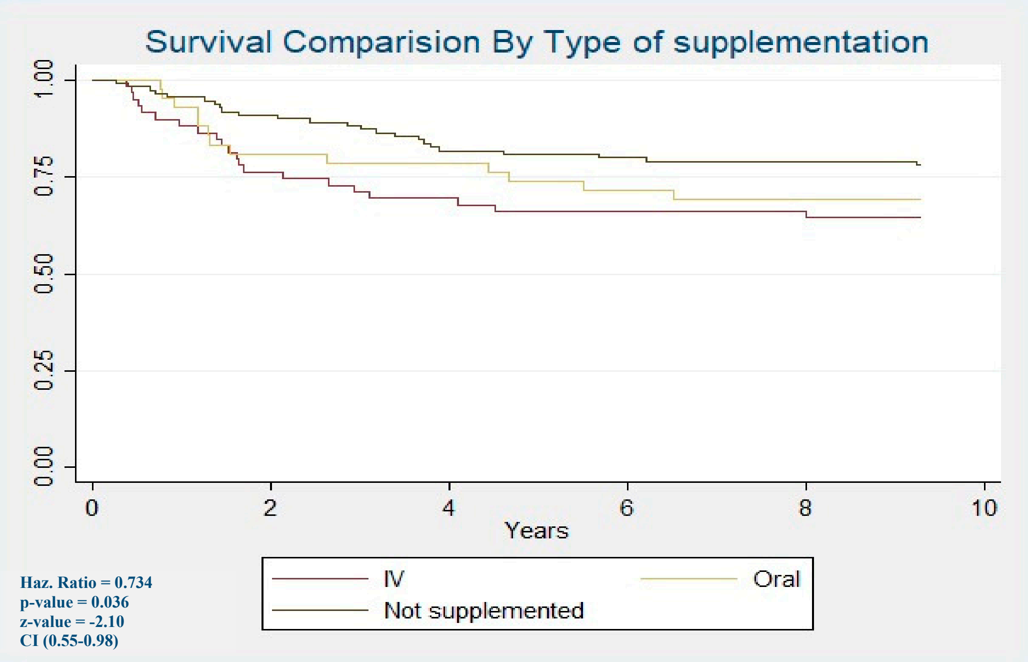

**Supplementary Figure S5**. Kaplan–Meier survival curves comparing overall survival according to type of iron supplementation in patients with heart failure. Survival was compared among patients receiving intravenous (IV) iron, oral iron, or no iron supplementation. The x-axis represents follow-up time (years), and the y-axis represents cumulative overall survival probability.

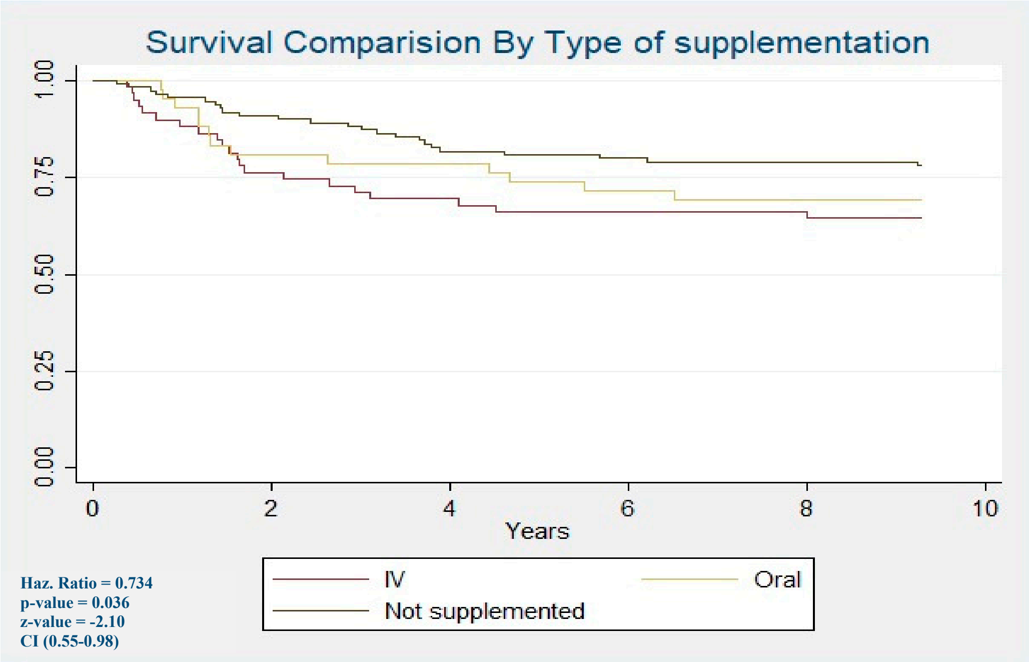


**Supplementary Figure S6 Love plot showing covariate balance before and after entropy balancing.**

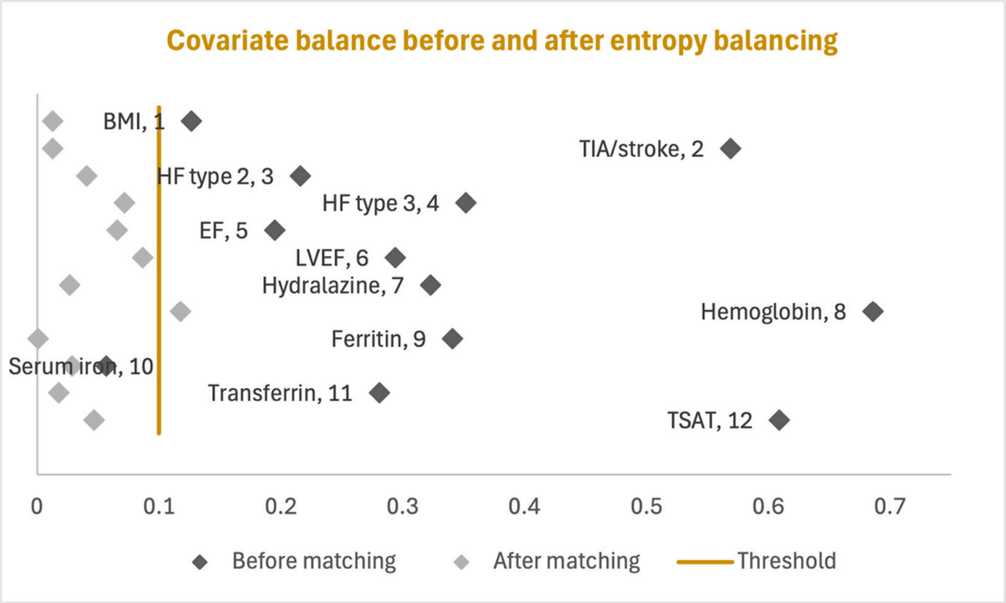


Absolute standardized mean differences are presented for the iron-supplemented and non-supplemented groups before and after entropy balancing. The dashed vertical line represents the conventional balance threshold of 0.10.

**Supplementary Table S1.** Baseline demographic, clinical, laboratory, and treatment characteristics of the study population according to the AHA-defined iron deficiency (ID) status in patients with heart failure.

| Category | AHA-HF ID definition | | | | |
|---|---|---|---|---|---|
| | **Overall** | **Untreated-ID** | **Treated ID** | **Not ID** | **p** |
| | **n =211** | **n = 61** | **n =83** | **n = 67** | |
| Duration of follow-up, IQR, year (*) | 1 (0.5-1.8) | 1.11 (0.6-2.2) | 1 (0.5-1.5) | 1.1 (0.5-2.5) | 0.295 |
| **Patient history (n, %)** | | | | | |
| Age (years) (mean, SD) (†) | 69.9±12.7 | 68.5±12.4 | 72.3±12.2 | 68.2±13.4 | 0.093 |
| Female | 122 (57.8) | 40 (65.6) | 50 (60.2) | 32 (47.8) | 0.106 |
| **History of cardiovascular disease (n, %)** | | | | | |
| IHD | 53 (25.1) | 10 (16.4) | 21 (25.3) | 22 (32.8) | 0.101 |
| PVD | 2 (0.95) | 0 (0.00) | 1 (1.20) | 1 (1.49) | 1.000 |
| AF (Fibrillation) | 58 (27.5) | 15 (24.6) | 26 (31.3) | 17 (25.4) | 0.600 |
| CAD | 42 (19.9) | 12 (19.7) | 16 (19.3) | 14 (20.9) | 0.969 |
| HTN | 173 (82.0) | 51 (83.6) | 73 (88.0) | 49 (73.1) | 0.059 |
| MI | 23 (10.9) | 6 (9.9) | 9 (10.8) | 8 (11.9) | 0.930 |
| **History (n, %)** | | | | | |
| CKD, Renal failure | 123 (58.3) | 31 (50.8) | 54 (65.1) | 38 (56.7) | 0.219 |
| Dyslipidemia | 52 (24.6) | 17 (27.9) | 22 (26.5) | 13 (19.4) | 0.475 |
| DM | 141 (66.8) | 43 (70.5) | 59 (71.1) | 39 (58.2) | 0.193 |
| Active Smoker | 9 (4.3) | 2 (3.3) | 4 (4.8) | 3 (4.5) | 1.000 |
| Alcoholism | 4 (1.9) | 1 (1.6) | 2 (2.4) | 1 (1.5) | 1.000 |
| Obesity (BMI > 30 kg/m²) (median, IQR) (*) | 28.9 (24.1-31.2) | 28.7 (24.5-32.1) | 28.9 (25.1-32.5) | 27.3 (22.8-29.5) | **0.017** |
| COPD | 7 (3.3) | 2 (3.3) | 3 (3.6) | 2 (3.0) | 1.000 |
| Depression | 7 (3.3) | 2 (3.3) | 4 (4.8) | 1 (1.5) | 0.564 |
| **Device therapy (n, %)** | | | | | |
| ICD | 2 (1.00) | 2 (3.3) | 0 (0.00) | 0 (0.00) | 0.083 |
| Pacemaker | 6 (2.8) | 2 (3.3) | 2 (2.4) | 2 (3.00) | 1.000 |
| Hypothyroidism | 19 (9.00) | 8 (13.1) | 8 (9.6) | 3 (4.5) | 0.225 |
| Malignancy | 9 (4.3) | 3 (4.9) | 3 (3.6) | 3 (4.5) | 0.916 |
| Stroke/ TIA | 21 (10.0) | 6 (9.8) | 2 (2.4) | 13 (19.4) | **<0.01** |
| Anemia | 197 (93.4) | 54 (88.5) | 81 (97.6) | 62 (92.5) | 0.079 |
| **Type of HF (n, %)** | | | | | |
| HFrEF | 65 (30.8) | 20 (32.8) | 20 (24.1) | 25 (37.3) | 0.202 |
| HFmidEF | 31 (14.7) | 9 (14.8) | 7 (8.4) | 15 (22.4) | 0.056 |
| HFpEF | 115 (54.5) | 32 (52.5) | 56 (67.5) | 27 (40.3) | **<0.01** |
| **Clinical characteristics (n, %) FC (NYHA)** | | | | | |
| I | 1 (3.9) | 0 (0.00) | 0 (0.00) | 1 (12.5) | 0.615 |
| II | 14 (53.9) | 5 (50.0) | 4 (50.0) | 5 (62.5) | 0.893 |
| III | 8 (30.8) | 4 (40.0) | 3 (37.5) | 1 (12.5) | 0.563 |
| IV | 3 (11.5) | 1 (10.0) | 1 (12.5) | 1 (12.5) | 1.000 |
| **Cause of HF (n, %)** | | | | | |
| Ischemic | **23 (10.9)** | **8 (13.1)** | **7 (8.4)** | **8 (11.9)** | **0.637** |
| Dilated Cardiomyopathy | **13 (6.2)** | **4 (6.6)** | **4 (4.8)** | **5 (7.5)** | **0.770** |
| Unknown | **175 (82.9)** | **49 (80.3)** | **72 (86.8)** | **54 (80.6)** | **0.496** |
| **Echocardiography (n, %)** | | | | | |

| | | | | | |
|---|---|---|---|---|---|
| EF (†) | 50 (35-60) | 50 (30-60) | 55 (40-60) | 45 (35-56) | 0.099 |
| LVEF (†) | 48.4±14.4 | 47.4±14.2 | 51.9±14.1 | 44.9±14.0 | **<0.01** |
| **Supplementation (n, %)** | | | | | |
| Iron therapy | 101 (47.9) | 0 (0.00) | 83 (100) | 18 (26.9) | **<0.01** |
| **Medication History (n, %)** | | | | | |
| ACEI | 59 (28.0) | 16 (26.2) | 20 (24.1) | 23 (34.3) | 0.358 |
| ARB | 45 (21.3) | 19 (31.2) | 15 (18.1) | 11 (16.4) | 0.082 |
| BB | 182 (86.3) | 55 (90.2) | 68 (81.9) | 59 (88.1) | 0.320 |
| Sacubitril / Valsartan | 4 (1.90) | 0 (0.00) | 1 (1.20) | 3 (4.5) | 0.192 |
| Amiodarone | 5 (2.4) | 1 (1.6) | 2 (2.4) | 2 (3.00) | 1.000 |
| CCB | 78 (37.0) | 22 (36.1) | 35 (42.2) | 21 (31.3) | 0.388 |
| Isosorbide | 45 (21.3) | 14 (23.0) | 20 (24.1) | 11 (16.4) | 0.487 |
| Hydralazine | 53 (25.1) | 13 (21.3) | 30 (36.1) | 10 (14.9) | **<0.01** |
| Digoxin | 10 (4.7) | 3 (4.9) | 4 (4.8) | 3 (4.5) | 1.000 |
| Statins | 158 (74.9) | 45 (73.8) | 62 (74.7) | 51 (76.1) | 0.953 |
| Diuretics | 2 (1.00) | 0 (0.00) | 1 (1.2) | 1 (1.5) | 1.000 |
| Thiazides | 14 (6.6) | 5 (8.2) | 5 (6.0) | 4 (6.0) | 0.884 |
| Anticoagulants | 55 (26.1) | 12 (19.7) | 27 (32.5) | 16 (23.9) | 0.196 |
| SGLT2 (dapaglphlozin) | 33 (15.6) | 11 (18.0) | 13 (15.7) | 9 (13.4) | 0.774 |
| NSAIDs | 1 (0.5) | 1 (1.6) | 0 (0.00) | 0 (0.00) | 0.289 |
| Asprin | 105 (49.8) | 35 (57.4) | 39 (47.0) | 31 (46.3) | 0.368 |
| Clopidogril | 48 (22.8) | 16 (26.2) | 17 (20.5) | 15 (22.4) | 0.716 |
| PPI | 114 (54.0) | 33 (54.1) | 52 (62.7) | 29 (43.3) | 0.061 |
| Spironolactone | 33 (15.6) | 8 (13.1) | 12 (14.5) | 13 (19.4) | 0.576 |
| Frusmide | 196 (92.9) | 57 (93.4) | 78 (94.0) | 61 (91.0) | 0.770 |
| Dose of Furusmide | 80 (40-80) | 80 (40-80) | 80 (40-80) | 40 (40-80) | 0.064 |
| **Baseline Analytical and Pharmacological Data** | | | | | |
| eGFR (mL/min/1.73 m²) (‡) | 53 (37-81) | 60 (40-83) | 49 (33-69) | 60 (40-80) | 0.100 |
| Creatinine (mg/dL) (‡) | 100 (72-137) | 96 (70-127) | 114 (72-146) | 96 (72-125) | 0.336 |
| CRP (mg/L) (‡) | 25 (6-76) | 28 (7-66) | 14 (4-63) | 39 (6-91) | 0.239 |
| NT-proBNP (pg/mL) (‡) | 2574 (1182-6263) | 1936.5 (888-6263) | 3226 (1411-8211) | 2918.5 (1263-5286) | 0.513 |
| SBP (mmHg) (†) | 132.4±24.3 | 132.8±23.2 | 134.5±23.9 | 129.3±25.9 | 0.424 |
| DBP (mmHg) (†) | 68.5±13.1 | 68.2±13.1 | 69.7±13.1 | 67.2±13.2 | 0.524 |
| Hemoglobin (g/dL) (†) | 9.5±1.8 | 9.9±1.8 | 9.0±1.6 | 9.8±1.9 | **<0.01** |
| Ferritin (ug/L) (‡) | 123.5 (42-282) | 68.5 (29-135) | 74 (26-142) | 406 (269-530) | **<0.01** |
| Iron (umol/L) (‡) | 6 (4-9) | 6 (5-8) | 4 (3-7_ | 10 (6-14) | **<0.01** |
| Transferrin (g/L) (†) | 2.2±0.7 | 2.3±0.6 | 2.4±0.6 | 1.7±0.6 | **<0.01** |
| TSAT (%) (‡) | 12.1 (7.4-19.9) | 10.8 (7.9-15.4) | 7.5 (5.2-12.4) | 23.4 (18.8-28.2) | **<0.01** |

Data are presented as mean ± standard deviation (SD) for normally distributed continuous variables, median (interquartile range [IQR]) for non-normally distributed continuous variables, and number (percentage) for categorical variables. Patients were classified into three groups according to the American Heart Association (AHA) definition of iron deficiency: untreated ID, treated ID, and non-ID. P-values compare differences among the three groups. Variables marked with (*) were analyzed as continuous variables. Abbreviations: ACEI, angiotensin-converting enzyme inhibitor; AF, atrial fibrillation; ARB, angiotensin receptor blocker; BB, beta-blocker; BMI, body mass index; CAD, coronary artery disease; CCB, calcium channel blocker; CKD, chronic

kidney disease; COPD, chronic obstructive pulmonary disease; CRP, C-reactive protein; DBP, diastolic blood pressure; DM, diabetes mellitus; EF, ejection fraction; eGFR, estimated glomerular filtration rate; HF, heart failure; HFmidEF, heart failure with mildly reduced ejection fraction; HFpEF, heart failure with preserved ejection fraction; HFrEF, heart failure with reduced ejection fraction; HTN, hypertension; ICD, implantable cardioverter-defibrillator; IHD, ischemic heart disease; IQR, interquartile range; LVEF, left ventricular ejection fraction; MI, myocardial infarction; NSAIDs, nonsteroidal anti-inflammatory drugs; NT-proBNP, N-terminal pro-B-type natriuretic peptide; NYHA, New York Heart Association; PPI, proton pump inhibitor; PVD, peripheral vascular disease; SBP, systolic blood pressure; SD, standard deviation; SGLT2, sodium-glucose cotransporter-2 inhibitor; TIA, transient ischemic attack; TSAT, transferrin saturation.

**Supplementary Table S2**. Selected clinical history, device therapy, and medication characteristics of the study population according to ESC-defined iron deficiency status in patients with heart failure.

| Category | ESC-HF ID definition | | | | |
|---|---|---|---|---|---|
| | **Overall** | **Untreated ID** | **Treated ID** | **Not ID** | **p** |
| | **n =211** | **n = 65** | **n =85** | **n = 61** | |
| **History (n, %)** | | | | | |
| Active Smoker | 9 (4.3) | 2 (3.1) | 4 (4.7) | 3 (4.9) | 0.835 |
| Alcoholism | 4 (1.9) | 1 (1.5) | 2 (2.4) | 1 (1.6) | 1.000 |
| COPD | 7 (3.3) | 2 (3.1) | 3 (3.5) | 2 (3.3) | 1.000 |
| Depression | 7 (3.3) | 2 (3.1) | 4 (4.7) | 1 (1.6) | 0.643 |
| **Device therapy (n, %)** | | | | | |
| ICD | 2 (1.00) | 2 (3.1) | 0 (0.0) | 0 (0.0) | 0.176 |
| Pacemaker | 6 (2.8) | 2 (3.1) | 2 (2.4) | 2 (3.3) | 1.000 |
| Hypothyroidism | 19 (9.00) | 8 (12.3) | 8 (9.4) | 3 (4.9) | 0.350 |
| Malignancy | 9 (4.3) | 3 (4.6) | 3 (3.5) | 3 (4.9) | 0.916 |
| **Medication History (n, %)** | | | | | |
| ACEI | 59 (28.0) | 16 (24.6) | 20 (23.5) | 23 (37.7) | 0.131 |
| ARB | 45 (21.3) | 20 (30.8) | 15 (17.7) | 10 (16.4) | 0.081 |
| BB | 182 (86.3) | 59 (90.8) | 70 (82.4) | 53 (86.9) | 0.328 |
| Sacubitril / Valsartan | 4 (1.90) | 0 (0.0) | 1 (1.2) | 3 (4.9) | 0.141 |
| Amiodarone | 5 (2.4) | 1 (1.5) | 2 (2.4) | 2 (3.3) | 0.864 |
| CCB | 78 (37.0) | 23 (35.4) | 37 (43.5) | 18 (29.5) | 0.213 |
| Isosorbide | 45 (21.3) | 14 (21.5) | 22 (25.9) | 9 (14.8) | 0.269 |
| Hydralazine | 53 (25.1) | 14 (21.5) | 31 (36.5) | 8 (13.1) | **<0.01** |
| Digoxin | 10 (4.7) | 3 (4.6) | 4 (4.7) | 3 (4.9) | 1.000 |
| Statins | 158 (74.9) | 49 (75.4) | 64 (75.3) | 45 (73.8) | 0.972 |
| Diuretics | 2 (1.00) | 0 (0.0) | 1 (1.2) | 1 (1.6) | 0.751 |
| Thiazides | 14 (6,6) | 6 (9.2) | 5 (5.9) | 3 (4.9) | 0.608 |
| Anticoagulants | 55 (26.1) | 12 (18.5) | 28 (32.9) | 15 (24.6) | 0.128 |
| SGLT2 (dapaglphlozin) | 33 (15.6) | 11 (16.9) | 13 (15.3) | 9 (14.8) | 0.939 |
| NSAIDs | 1 (0.5) | 1 (1.5) | 0 (0.0) | 0 (0.0) | 0.597 |
| Asprin | 105 (49.8) | 38 (58.5) | 41 (48.2) | 26 (42.6) | 0.193 |
| Clopidogril | 48 (22.8) | 16 (24.6) | 18 (21.2) | 14 (23.0) | 0.883 |
| PPI | 114 (54.0) | 35 (53.9) | 53 (62.4) | 26 (42.6) | 0.062 |

| | | | | | |
|---|---|---|---|---|---|
| Spironolactone | 33 (15.6) | 9 (13.9) | 12 (14.1) | 12 (19.7) | 0.589 |
| Frusmide | 196 (92.9) | 60 (92.3) | 79 (92.9) | 57 (93.4) | 0.970 |
| Dose of Furusmide | 80 (40-80) | 80 (40-80) | 80 (40-80) | 40 (40-80) | 0.155 |

Data are presented as number (percentage) for categorical variables and median (interquartile range [IQR]) for furosemide dose. Patients were classified according to the European Society of Cardiology (ESC) definition of iron deficiency into untreated iron deficiency (ID), treated ID, and non-ID groups. P-values represent comparisons among the three groups. ACEI, angiotensin-converting enzyme inhibitor; ARB, angiotensin receptor blocker; BB, beta-blocker; CCB, calcium channel blocker; COPD, chronic obstructive pulmonary disease; ESC, European Society of Cardiology; HF, heart failure; ICD, implantable cardioverter-defibrillator; ID, iron deficiency; NSAIDs, nonsteroidal anti-inflammatory drugs; PPI, proton pump inhibitor; SGLT2, sodium–glucose cotransporter-2 inhibitor.

**Supplementary Table S3. Changes in biochemical, echocardiographic, and clinical parameters according to AHA- and ESC-defined iron deficiency status in patients with heart failure.**

| Category | AHA-HF ID definition | | | | | ESC-HF ID definition | | | | |
|---|---|---|---|---|---|---|---|---|---|---|
| | **Total number** | **Untreated ID** | **Treated ID** | **Not ID** | **p-value** | **Total number** | **Untreated ID** | **Treated ID** | **Not ID** | **p-value** |
| | **n=211** | **n=63** | **n=60** | **n=88** | | **n=211** | **n=66** | **n=63** | **n=82** | |
| **Biochemical change** | | | | | | | | | | |
| **Absolute Change of Analytical Data** | | | | | | | | | | |
| Absolute Change EF (%) (*) | 0 (-1-2) | 0 (-3-0) | 0 (-5-0.5) | 0 (0-4) | 0.088 | 0 (-1-2) | 0 (-3-0) | 0 (-5-1) | 0 (0-4) | 0.127 |
| Absolute Change LVEF (%) (*) | 0.5 (-6-6) | -1.7 (-7-3) | 1 (-8-6) | 1.5 (-3-8) | 0.411 | 0.5 (-6-6) | -1.8 (-7-2.5) | 1 (-5.5-6) | 1 (-3-8) | 0.309 |
| Absolute Change eGFR (mL/min/1.73 m²) (*) | -3 (-14-4) | 0 (-15-7) | -2.5 (-14.5-2) | -4 (-13.5-3.5) | 0.824 | -3 (-14-4) | -1 (-15-5) | -3 (-15-2) | -4 (-13-4) | 0.873 |
| Absolute Change Creatinine (mg/dL) (*) | 8 (-10-31) | 5 (-11-28) | 4.5 (-5.5-54) | 14 (-12-28.5) | 0.855 | 8 (-10-31) | 6.5 (-11-28) | 5 (-6-56) | 12.5 (-12-27) | 0.908 |
| Absolute Change CRP (mg/L) (*) | 0 (-28-32) | 0 (-23-27) | 3 (-10-50) | -2 (-44-30) | 0.430 | 0 (-28-32) | 0 (-23-38) | 2 (-7.5-41) | -5.5 (-45-26) | 0.389 |
| Absolute Change NT-ProBNP (pg/mL) (*) | 797 (-312-4287) | 620 (-856-2061) | 208 (-2773-4287) | 1266 (-34-12971) | 0.215 | 797 (-312-4287) | 455 (-856-2061) | 208 (-1841.5-3540) | 1556 (234-13052) | 0.131 |
| Absolute Change SBP (mmHg) (*) | -4.3±25.6 | -1.0±25.6 | -5.1±26.8 | -6.1±27.8 | 0.476 | -4.3±25.6 | -2.2±27.4 | -5.9±27.3 | -4.7±22.7 | 0.703 |
| Absolute Change DBP (mmHg) (*) | 0.04±17.2 | 1.2±19.1 | 1.8±16.8 | -2.0±16.1 | 0.352 | 0.04±17.2 | 0.8±19.1 | 0.8±17.1 | -1.2±15.8 | 0.695 |
| Absolute Change Hemoglobin (g/dL) (*) | 0.01±2.0 | -.04±2.0 | 0.3±2.1 | 0.1±1.9 | 0.085 | 0.01±2.0 | -0.4±1.9 | 0.2±2.1 | 0.2±1.9 | 0.085 |
| Absolute Change Ferritin (ug/L) (*) | 5 (-58-85) | -33 (-93-6) | 9 (-22-42) | 78 (-39-385) | **<0.01** | 5 (-58-85) | -30 (-86-25) | 17 (-22-66) | 41 (-74-421) | **0.037** |
| Absolute Change Iron (umol/L) (*) | 1 (-2-4) | 0 (-3-1) | 1 (-1-2) | 4 (0-7) | **<0.01** | 1 (-2-4) | -1 (-3-1) | 1 (-1-2) | 4 (0-7) | **<0.01** |
| Absolute Change Transferrin (g/L) (*) | -0.1 (-.04-0.3) | 0.34 (-0.3-0.7) | -0.2 (-0.6-0.2) | -0.1 (-0.5-0.1) | **<0.01** | -0.1 (-.04-0.3) | 0.3 (-0.3-0.6) | -0.2 (-0.6-0.1) | -0.1 (-0.5-0.1) | **<0.01** |

| | | | | | | | | | | |
|---|---|---|---|---|---|---|---|---|---|---|
| Absolute Change TSAT (%) (*) | 1.9 (-2-9.4) | -1.7 (-8.2-1.6) | 1.4 (-1-4.5) | 10.5 (1.7-19.4) | **<0.01** | 1.9 (-2-9.4) | -1.8 (-8-1.6) | 1.4 (-1-4.3) | 11.3 (2.4-19.8) | **<0.01** |

Absolute changes were calculated as the follow-up value minus the baseline value. Data are presented as mean ± standard deviation (SD) for normally distributed variables and median (interquartile range [IQR]) for non-normally distributed variables. Patients were classified as having untreated iron deficiency (ID), treated ID, or no ID according to the American Heart Association (AHA) and European Society of Cardiology (ESC) definitions. P-values

## Supplementary Table S4 Changes in echocardiographic, biochemical, and clinical parameters according to iron supplementation status in patients with heart failure.

| Category | Total number n=211 | Supplemented n=101 | Not Supplemented n=110 | p-value |
|---|---|---|---|---|
| **Biochemical change** | | | | |
| **Absolute Change of Analytical Data** | | | | |
| Absolute Change EF (%) (*) | 0 (-1-2) | 0 (-4-1) | 0 (0-3) | 0.257 |
| Absolute Change LVEF (%) (*) | 0.5 (-6-6) | 1 (-4-6) | 0 (-7-5) | 0.633 |
| Absolute Change eGFR (mL/min/1.73 m²) (*) | -3 (-14-4) | -4 (-14-1) | -0.5 (-13-5) | 0.230 |
| Absolute Change Creatinine (mg/dL) (*) | 8 (-10-31) | 11 (-6-56) | 3.5 (-11-26) | 0.224 |
| Absolute Change CRP (mg/L) (*) | 0 (-28-32) | 0 (-22-17) | 0 (-34.5-40.5) | 0.649 |
| Absolute Change NT-ProBNP (pg/mL) (*) | 797 (-312-4287) | 423 (-459-3950) | 808 (-197-6064.5) | 0.747 |
| Absolute Change SBP (mmHg) (*) | -4.3±25.6 | -5.8±25.4 | -2.9±25.8 | 0.416 |
| Absolute Change DBP (mmHg) (*) | 0.04±17.2 | -0.5±17.5 | 0.6±17.0 | 0.618 |
| Absolute Change Hemoglobin (g/dL) (*) | 0.01±2.0 | 0.2±2.0 | -0.2±1.9 | 0.077 |
| Absolute Change Ferritin (ug/L) (*) | 5 (-58-85) | 32 (-22-170) | -30 (-93-40) | **<0.01** |
| Absolute Change Iron (umol/L) (*) | 1 (-2-4) | 1 (-1-5) | 0 (-2-4) | 0.050 |
| Absolute Change Transferrin (g/L) (*) | -0.1 (-0.4-0.3) | -0.2 (-0.6-0.1) | 0.1 (-0.3-0.5) | **<0.01** |
| Absolute Change TSAT (%) (*) | 1.9 (-2-9.4) | 2.8 (-0.3-11.1) | 0.1 (-5.6-8.3) | **<0.01** |
| Supplementation Cumulative Dose (*) | 0 (0-5760) | 5760 (1488-15360) | 0 (0-0) | **<0.01** |

Absolute changes were calculated as the follow-up value minus the baseline value. Data are presented as mean ± standard deviation (SD) for normally distributed variables and median (interquartile range [IQR]) for non-normally distributed variables. Patients were classified into supplemented and non-supplemented groups. P-values represent comparisons between the two groups. Variables marked with an asterisk (*) were analyzed as continuous variables.

**Supplementary Table S5. Clinical outcomes according to intravenous iron formulation among iron-supplemented patients with heart failure.**

| Category | Total number n=59 | Supplemented IV=59 | | | p-value |
|---|---|---|---|---|---|
| | | Saccharate | Iron Sucrose | Ferric Carboxymaltose (FCM) | |
| | | n=16 | n=12 | n=29 | |
| **Emergency visits (n, %)** | | | | | |
| Total Emg | 58 (98.3) | 16 (100) | 13 (92.9) | 29 (100) | 0.237 |
| HF-Emg | 40 (67.8) | 15 (93.8) | 8 (57.1) | 17 (58.6) | **0.024** |
| CVD-emg | 20(33.9) | 7 (43.8) | 4 (28.6) | 9 (31.0) | 0.676 |
| **Admission (n, %)** | | | | | |
| Total admission | 57 (96.6) | 16 (100) | 12 (85.7) | 29 (100) | 0.053 |
| HF-admission | 38 (64.4) | 14 (87.5) | 7 (50.0) | 17 (58.6) | 0.071 |
| CVD- admission | 26 (44.1) | 10 (62.5) | 5 (35.7) | 11 (37.9) | 0.218 |
| **All-cause mortality (n, %)** | 21 (35.6) | 9 (56.3) | 5 (35.7) | 7 (24.1) | 0.098 |
| HF | 5 (8.5) | 3 (18.8) | 0 (0.0) | 2 (6.9) | 0.223 |
| CVD | 7 (11.9) | 2 (12.5) | 3 (21.4) | 2 (6.9) | 0.380 |
| Cardiovascular death (HF & CVD combined | 12 (20.3) | 5 (31.3) | 3 (21.4) | 4 (13.8) | 0.377 |
| Other | 9 (15.3) | 4 (25.0) | 2 (14.3) | 3 (10.3) | 0.411 |
| **CVD occurrence (n, %)** | | | | | |
| IHD | 0 (0.0) | 0 (0.0) | 0 (0.0) | 0 (0.0) | - |
| Stroke | 4 (6.8) | 1 (6.3) | 0 (0.0) | 3 (10.3) | 0.800 |
| MI | 7 (11.9) | 2 (12.5) | 2 (14.3) | 3 (10.3) | 1.000 |
| TIA | 1 (1.7) | 0 (0.0) | 0 (0.0) | 1 (3.5) | 1.000 |
| AF | 21 (35.6) | 6 (37.5) | 5 (35.7) | 10 (34.5) | 0.980 |

Data are presented as number (percentage). The analysis includes only patients who received intravenous (IV) iron supplementation and compares outcomes among those treated with ferric saccharate, iron sucrose, and ferric carboxymaltose (FCM). P-values represent comparisons among the three IV iron formulations.

**Supplementary Table S*6*** Comparison of Clinical Adverse Outcomes Between Iron Deficiency (ID), Treated Iron Deficiency (Treated ID), and Non-Iron Deficiency (Non-ID) Groups in Patients with Heart Failure with Reduced Ejection Fraction (HFrEF)

| Category | AHA-HF ID definition | | | | | ESC-HF ID definition | | | | |
|---|---|---|---|---|---|---|---|---|---|---|
| | Total number | Untreated ID | Treated ID | Not ID | p-value | Total number | Untreated ID | Treated ID | Not ID | p-value |
| | n=65 | n=18 | n=15 | n=32 | | n=65 | n=19 | n=15 | n=31 | |
| **Clinical adverse outcome** | | | | | | | | | | |
| **Emergency visits (n, %)** | | | | | | | | | | |
| Total Emg | 60 (92.3) | 16 (88.9) | 15 (100) | 29 (90.6) | 0.587 | 60 (92.3) | 17 (89.5) | 15 (100) | 28 (90.3) | 0.588 |
| Total number of Emg (*) | 3(1-6) | 3 (1-6) | 3(2-7) | 3 (1-5) | 0.799 | 3(1-6) | 3 (1-7) | 3 (2-7) | 3 (1-5) | 0.737 |
| HF-Emg | 31 (47.7) | 12 (66.7) | 7 (46.7) | 12 (37.5) | 0.140 | 31 (47.7) | 12 (63.2) | 7 (46.7) | 12 (38.7) | 0.243 |
| CVD-emg | 12 (18.5) | 5 (27.8) | 1 (6.7) | 6 (18.8) | 0.313 | 12 (18.5) | 5 (26.3) | 1 (6.7) | 6 (19.4) | 0.348 |
| **Admission (n, %)** | | | | | | | | | | |
| Total- admission | 54 (83.1) | 15 (83.3) | 14 (93.3) | 25 (78.1) | 0.477 | 54 (83.1) | 16 (84.2) | 14 (93.3) | 24 (77.4) | 0.395 |
| Length of hospital stay (days) (*) | 2 (1-3) | 2 (2-3) | 3 (2-4) | 2 (1-3.5) | 0.393 | 2 (1-3) | 2 (2-3) | 3 (2-4) | 2 (1-4) | 0.411 |
| HF-admission | 34 (52.3) | 13 (72.2) | 7 (46.7) | 14 (43.8) | 0.136 | 34 (52.3) | 14 (73.7) | 7 (46.7) | 13 (41.9) | 0.082 |
| CVD- admission | 20 (30.8) | 6 (33.3) | 4 (26.7) | 10 (31.3) | 1.000 | 20 (30.8) | 6 (31.6) | 4 (26.7) | 10 (32.3) | 1.000 |
| **HF decompensation (n, %)** | | | | | | | | | | |
| Arrhythmia | 0 (0.0) | 0 (0.0) | 0 (0.0) | 0 (0.0) | - | 0 (0.0) | 0 (0.0) | 0 (0.0) | 0 (0.0) | - |
| Infection | 15 (23.1) | 4 (22.2) | 4 (26.7) | 7 (21.9) | 0.929 | 15 (23.1) | 4 (21.1) | 4 (26.7) | 7 (22.6) | 1.000 |
| Ischemic | 0 (0.0) | 0 (0.0) | 0 (0.0) | 0 (0.0) | - | 0 (0.0) | 0 (0.0) | 0 (0.0) | 0 (0.0) | - |
| Unknown | 19 (29.2) | 9 (50.0) | 3 (20.0) | 7 (21.9) | 0.104 | 19 (29.2) | 10 (52.6) | 3 (20.0) | 6 (19.4) | **0.038** |
| **All-cause mortality (n, %)** | 16 (24.6) | 4 (22.2) | 2 (26.7) | 8 (25.0) | 1.000 | 16 (24.6) | 4 (21.1) | 4 (26.7) | 8 (25.8) | 1.000 |
| **Reason of death (n, %)** | | | | | | | | | | |
| HF | 4 (6.2) | 2 (11.1) | 1 (6.7) | 1 (3.1) | 0.448 | 4 (6.2) | 2 (10.5) | 1 (6.7) | 1 (3.2) | 0.678 |
| CVD | 4 (6.2) | 0 (0.0) | 1 (6.7) | 3 (9.4) | 0.558 | 4 (6.2) | 0 (0.0) | 1 (6.7) | 3 (9.7) | 0.443 |
| Other | 8 (12.3) | 2 (11.1) | 2 (13.3) | 4 (12.5) | 1.000 | 8 (12.3) | 2 (10.5) | 2 (13.3) | 4 (12.9) | 1.000 |
| **Composite outcome (n, %)** | 61 (93.9) | 16 (88.9) | 15 (100) | 30(93.8) | 0.670 | 61 (93.9) | 17 (89.5) | 15(100) | 29 (93.6) | 0.678 |
| **CVD occurrence (n, %)** | | | | | | | | | | |
| IHD | 1 (1.5) | 1 (5.6) | 0 (0.0) | 0 (0.0) | 0.508 | 1 (1.5) | 1 (5.3) | 0 (0.0) | 0 (0.0) | 0.523 |
| Stroke | 8 (12.3) | 2 (11.1) | 2 (13.3) | 4 (12.5) | 1.000 | 8 (12.3) | 2 (10.5) | 2 (13.3) | 4 (12.9) | 1.000 |
| MI | 5 (7.7) | 4 (22.2) | 0 (0.0) | 1 (3.1) | 0.046 | 5 (7.7) | 4 (21.1) | 0 (0.0) | 1 (3.2) | 0.053 |
| TIA | 1 (1.5) | 1 (5.6) | 0 (0.0) | 0 (0.0) | 0.508 | 1 (1.5) | 1 (5.3) | 0 (0.0) | 0 (0.0) | 0.523 |
| AF | 8 (12.3) | 0 (0.0) | 2 (13.3) | 6 (18.8) | 0.132 | 8 (12.3) | 0 (0.0) | 2 (13.3) | 6 (19.2) | 0.116 |

| | | | | | | | | | | |
|---|---|---|---|---|---|---|---|---|---|---|
| <u>**Biochemical change**</u> | | | | | | | | | | |
| **Absolute Change of Analytical Data** | | | | | | | | | | |
| Absolute Change EF (%) (*) | 0 (0-0) | 0 (-5-0) | 0 (-2-0) | 0 (0-2.5) | 0.518 | 0 (0-0) | 0 (-5-0) | 0 (-2-0) | 0 (0-4) | 0.509 |
| Absolute Change LVEF (%) (*) | 0.5 (-7.5) | 0 (-7-2) | -0.5(-8-4) | 1 (-17-4) | 0.999 | 0.5 (-7.5) | -1 (-7.5-2) | -0.5(-8-4) | 1 (-3-4) | 0.819 |
| Absolute Change eGFR (mL/min/1.73 m²) (*) | -2 (-14-3) | 0 (-4-4) | -9(-17-2) | -3.5 (-16.5-3) | 0.311 | -2 (-14-3) | 0 (-4-4) | -9(-17-2) | -4(-18-3) | 0.235 |
| Absolute Change Creatinine (mg/dL) (*) | 4 (-12-33) | 1.5 (-15-19) | 11 (0-58) | 3 (-14-39) | 0.400 | 4 (-12-33) | 1 (-15-19) | 11 (0-58) | 4 (-15-60) | 0.346 |
| Absolute Change CRP (mg/L) (*) | -9.5 (-33-19) | -16 (-51- -9) | -28.5(-46- -10) | 0 (-18-48) | 0.073 | -9.5 (-33-19) | -12.5(-51-8) | -28.5(-46- -10) | -1 (-18-48) | 0.155 |
| Absolute Change NT-ProBNP (pg/mL) (*) | 1523.5 (-1413-9249) | 905 (-1413-7203) | 4381.5 (-5020-15910) | 1556 (-34-21248) | 0.937 | 1523.5 (-1413-9249) | 905 (-1413-7203) | 4381.5 (-5020-15910) | 1556 (-34-21248) | 0.937 |
| Absolute Change SBP (mmHg) (*) | -2.9±20.3 | -2.2±24.4 | -7.7±18.0 | -0.9±19.0 | 0.568 | -2.9±20.3 | -1.7±23.8 | -7.7±18.0 | -1.2±19.3 | 0.578 |
| Absolute Change DBP (mmHg) (*) | -0.4±15.9 | -1.7±12.7 | 2.8±19.4 | -1.1±16.2 | 0.678 | -0.4±15.9 | -1.2±12.6 | 2.8±19.4 | -1.5±16.4 | 0.681 |
| Absolute Change Hemoglobin (g/dL) (*) | -0.2±1.9 | -0.6±2.0 | -0.02±2.3 | -0.1±1.9 | 0.666 | -0.2±1.9 | -0.6±1.9 | -0.02±2.3 | -0.1±1.9 | 0.611 |
| Absolute Change Ferritin (ug/L) (*) | -28(-93-85) | -49(-93- -33) | 50 (3-85) | 41 (-68.5-345.5) | 0.047 | -28 (-93-85) | -44.5 (-93- -32) | 50 (3-85) | 13 (-98-85) | 0.130 |
| Absolute Change Iron (umol/L) (*) | 1 (-1-4.5) | 0 (-3-2) | 1 (-2-2) | 4 (-1-6) | 0.058 | 1 (-1-4.5) | 0 (-3-2) | 1 (-2-2) | 4 (-1-6) | **0.029** |
| Absolute Change Transferrin (g/L) (*) | 0 (-0.4-0.3) | 0.4 (-0.1-1.2) | -0.4 (-0.7-0.2) | -0.04(-0.5-0.1) | <0.01 | 0 (-0.4-0.3) | 0.3(-0.2-1.2) | -0.4(-0.7-0.2) | -0.03 (-0.5-0.1) | **<0.01** |
| Absolute Change TSAT (%) (*) | 2.7(-1.4-9.1) | -2.4(-14.8-2) | 2.4(0.2-5.1) | 7.7(2.5-16.9) | <0.01 | 2.7 (-1.4-9.1) | -2.3 (-14.8-2.4) | 2.4(0.2-5.1) | 8.3 (2.5-17.5) | **<0.01** |

Data are presented as number (percentage) for categorical variables, mean ± standard deviation (SD) for normally distributed continuous variables, and median (interquartile range [IQR]) for non-normally distributed continuous variables. Absolute changes were calculated as the follow-up value minus the baseline value. Patients were classified as having untreated iron deficiency (ID), treated ID, or no ID according to the American Heart Association (AHA) and European Society of Cardiology (ESC) definitions. P-values represent comparisons among the three groups within each definition. Variables marked with an asterisk (*) were analyzed as continuous variables.

**Supplementary Table S7** Comparison of Clinical Adverse Outcomes Between Iron Deficiency (ID) and Non-Iron Deficiency (Non-ID) Groups in Patients with Heart Failure with Reduced Ejection Fraction (HFrEF)

| Category | AHA-HF ID definition | | | | ESC-HF ID definition | | | |
|---|---|---|---|---|---|---|---|---|
| | Total number | ID | Not ID | p-value | Total number | ID | Not ID | p-value |
| | n=65 | n=33 | n=32 | | n=65 | n=34 | n=31 | |
| **Clinical adverse outcome** | | | | | | | | |
| **Emergency visits (n, %)** | | | | | | | | |
| Total Emg | 60 (92.3) | 31 (93.9) | 29 (90.6) | 0.616 | 60 (92.3) | 32 (94.1) | 28 (90.3) | 0.566 |
| Total number of Emg (*) | 3 (1-6) | 3 (2-7) | 3 (1-5) | 0.653 | 3 (1-6) | 3 (1-5) | 3 (2-7) | 0.483 |
| HF-Emg | 31 (47.7) | 19 (57.6) | 12 (37.5) | 0.105 | 31 (47.7) | 19 (55.9) | 12 (38.7) | 0.166 |
| CVD-emg | 12 (18.5) | 6 (18.2) | 6 (18.8) | 0.953 | 12 (18.5) | 6 (17.7) | 6 (19.4) | 0.859 |
| **Admission (n, %)** | | | | | | | | |
| Total- admission | 54 (93.1) | 29 (87.9) | 25 (78.1) | 0.339 | 54 (93.1) | 30 (88.2) | 24 (77.4) | 0.327 |
| Length of hospital stay (days) (*) | 2 (1-3) | 2 (2-3) | 3 (1-3.5) | 0.663 | 2 (1-3) | 2 (2-3) | 2 (1-4) | 0.569 |
| HF-admission | 34 (52.3) | 20 (60.6) | 14 (43.8) | 0.174 | 34 (52.3) | 21 (61.8) | 13 (41.9) | 0.110 |
| CVD- admission | 20 (30.8) | 10 (30.3) | 10 (31.3) | 0.934 | 20 (30.8) | 10 (29.4) | 10 (32.3) | 0.804 |
| **HF decompensation (n, %)** | | | | | | | | |
| Arrhythmia | 0 (0.0) | 0 (0.0) | 0 (0.0) | - | 0 (0.0) | 0 (0.0) | 0 (0.0) | - |
| Infection | 15 (23.1) | 8 (24.2) | 7 (21.9) | 0.821 | 15 (23.1) | 8 (23.5) | 7 (22.6) | 0.928 |
| Ischemic | 0 (0.0) | 0 (0.0) | 0 (0.0) | - | 0 (0.0) | 0 (0.0) | 0 (0.0) | - |
| Unknown | 19 (29.2) | 12 (36.4) | 7 (21.9) | 0.199 | 19 (29.2) | 13 (38.2) | 6 (19.4) | 0.095 |
| **All-cause mortality (n, %)** | 16 (24.6) | 8 (24.2) | 8 (25.0) | 0.943 | 16 (24.6) | 8 (23.5) | 8 (25.8) | 0.831 |
| **Reason of death (n, %)** | | | | | | | | |
| HF | 4 (6.2) | 3 (9.1) | 1 (3.1) | 0.613 | 4 (6.2) | 3 (8.8) | 1 (3.2) | 0.615 |
| CVD | 4 (6.2) | 1 (3.0) | 3 (9.4) | 0.355 | 4 (6.2) | 1 (2.9) | 3 (9.7) | 0.341 |
| Other | 8 (12.3) | 4 (12.1) | 4 (12.5) | 1.000 | 8 (12.3) | 4 (11.8) | 4 (12.9) | 1.000 |
| **Composite outcome (n, %)** | 61 (93.9) | 31 (93.9) | 30 (93.8) | 1.000 | 61 (93.9) | 32 (94.1) | 29 (93.6) | 1.000 |
| **CVD occurrence (n, %)** | | | | | | | | |
| IHD | 1 (1.5) | 1 (3.0) | 0 (0.0) | 1.000 | 1 (1.5) | 1 (2.9) | 0 (0.0) | 1.000 |
| Stroke | 8 (12.3) | 4 (12.1) | 4 (12.5) | 1.000 | 8 (12.3) | 4 (11.8) | 4 (12.9) | 1.000 |
| MI | 5 (7.7) | 4 (12.1) | 1 (3.1) | 0.355 | 5 (7.7) | 4 (11.8) | 1 (3.2) | 0.358 |
| TIA | 1 (1.5) | 1 (3.0) | 0 (0.0) | 1.000 | 1 (1.5) | 1 (2.9) | 0 (0.0) | 1.000 |
| AF | 8 (12.3) | 2 (6.1) | 6 (18.8) | 0.149 | 8 (12.3) | 2 (5.9) | 6 (19.4) | 0.138 |
| **Biochemical change** | | | | | | | | |
| **Absolute Change of Analytical Data** | | | | | | | | |
| Absolute Change EF (%) (*) | 0 (0-0) | 0 (-2-0) | 0 (0-2.5) | 0.252 | 0 (0-0) | 0 (-2-0) | 0 (0-4) | 0.245 |
| Absolute Change LVEF (%) (*) | 0.5 (-7.5-3) | 0 (-7-2) | 1 (-17-4) | 0.969 | 0.5 (-7.5-3) | -1 (-8-2) | 1 (-3-4) | 0.567 |
| Absolute Change eGFR (mL/min/1.73 m²) (*) | -2 (-14-3) | -2 (-13-2) | -3.5 (-16.5-3) | 0.722 | -2 (-14-3) | -1.5 (-13-3) | -4 (-18-3) | 0.562 |

| | | | | | | | | |
|---|---|---|---|---|---|---|---|---|
| Absolute Change Creatinine (mg/dL) (*) | 4 (-12-33) | 5 (-6-33) | 3 (-14-39) | 0.849 | 4 (-12-33) | 4.5 (-8-33) | 4 (-15-60) | 0.984 |
| Absolute Change CRP (mg/L) (*) | -9.5 (-33-19) | -24 (-51- -9) | 0 (-18-48) | 0.022 | -9.5 (-33-19) | -20 (-48.5- -1) | -1 (-18-48) | 0.065 |
| Absolute Change NT-ProBNP (pg/mL) (*) | 1523.5 (-1413-9249) | 1491 (-5020-9249) | 1556 (-34-21248) | 0.775 | 1523.5 (-1413-9249) | 1491 (-5020-9249) | 1556 (-34-21248) | 0.775 |
| Absolute Change SBP (mmHg) (*) | -2.9±20.3 | -4.7±21.6 | -0.9±19.0 | 0..462 | -2.9±20.3 | -4.4±21.3 | -1.2±19.3 | 0.538 |
| Absolute Change DBP (mmHg) (*) | -0.4±15.9 | 0.3±15.9 | -1.1±16.2 | 0.708 | -0.4±15.9 | 0.6±15.8 | -1.5±16.4 | 0.604 |
| Absolute Change Hemoglobin (g/dL) (*) | -0.2±1.9 | -0.3±2.1 | -0.1±1.9 | 0.696 | -0.2±1.9 | -0.4±2.1 | -0.1±1.9 | 0.619 |
| Absolute Change Ferritin (ug/L) (*) | -28 (-93-85) | -33 (-93-34) | 41 (-68.5-343.5) | 0.130 | -28 (-93-85) | -32.5(-87.5-50) | 13 (-98-385) | 0.256 |
| Absolute Change Iron (umol/L) (*) | 1 (-1-4.5) | 0 (-2-2) | 4 (-1-6) | 0.018 | 1 (-1-4.5) | 0 (-2-2) | 4 (-1-6) | **<0.01** |
| Absolute Change Transferrin (g/L) (*) | 0 (-0.4-0.3) | 0.03(-0.4-0.4) | -0.03(-0.5-0.1) | 0.174 | 0 (-0.4-0.3) | 0.02(-0.4-0.4) | -0.3(-0.5-0.1) | 0.340 |
| Absolute Change TSAT (%) (*) | 2.7(-1.4-9.1) | 0.3(-4.5-4.3) | 7.7(2.5-16.9) | <0.01 | 2.7(-1.4-9.1) | 0.4 (-2.9-4.1) | 8.3(2.5-17.5) | **<0.01** |

Data are presented as number (percentage) for categorical variables, mean ± standard deviation (SD) for normally distributed continuous variables, and median (interquartile range [IQR]) for non-normally distributed continuous variables. Absolute changes were calculated as the follow-up value minus the baseline value. Iron-supplemented patients were classified as having iron deficiency (ID) or no ID according to the American Heart Association (AHA) and European Society of Cardiology (ESC) definitions. P-values represent comparisons between the ID and non-ID groups within each definition. Variables marked with an asterisk (*) were analyzed as continuous variables.

**Supplementary Table S8.** Comparison of Clinical Adverse Outcomes Between Iron Deficiency (ID), Treated Iron Deficiency (Treated ID), and Non-Iron Deficiency (Non-ID) Groups in Patients with Heart Failure with Preserved Ejection Fraction (HFpEF)

| Category | AHA-HF ID definition | | | | | ESC-HF ID definition | | | | |
|---|---|---|---|---|---|---|---|---|---|---|
| | Total number | Untreated ID | Treated ID | Not ID | p-value | Total number | Untreated ID | Treated ID | Not ID | p-value |
| | n=115 | n=33 | n=39 | n=43 | | n=115 | n=35 | n=41 | n=39 | |
| **Clinical adverse outcome** | | | | | | | | | | |
| **Emergency visits (n, %)** | | | | | | | | | | |
| Total Emg | 107 (93.0) | 32 (97.0) | 37 (94.9) | 38 (88.4) | 0.381 | 107 (93.0) | 34 (97.1) | 39 (95.1) | 34 (87.2) | 0.215 |
| Total number of Emg (*) | 3 (2-6) | 3(2-6) | 2 (2-5) | 3 (1-6) | 0.761 | 3 (2-6) | 3(2-6) | 3 (2-5) | 3 (1-6) | 0.876 |
| HF-Emg | 78 (67.8) | 27 (81.8) | 26 (66.7) | 25 (58.1) | 0.089 | 78 (67.8) | 27 (77.1) | 28 (68.3) | 23 (59.0) | 0.247 |
| CVD-emg | 28 (24.4) | 8 (24.2) | 12 (30.8) | 8 (18.6) | 0.440 | 28 (24.4) | 8 (22.9) | 13 (31.7) | 7 (18.0) | 0.347 |
| **Admission (n, %)** | | | | | | | | | | |
| Total- admission | 104 (90.4) | 31(93.9) | 38 (97.4) | 35(81.4) | 0.043 | 104 (90.4) | 32 (91.4) | 40(97.6) | 32(82.1) | 0.062 |
| Length of hospital stay (days) (*) | 2 (1-4) | 2 (1-3) | 2 (2-4) | 2 (1-4) | 0.609 | 2 (1-4) | 2 (1-3) | 2 (2-4) | 2 (1-4) | 0.327 |
| HF-admission | 72(62.6) | 23(69.7) | 25(64.1) | 24(55.8) | 0.451 | 72(62.6) | 23 (65.7) | 27(65.9) | 22(56.4) | 0.616 |
| CVD- admission | 36 (31.3) | 9 (27.3) | 18 (46.2) | 9 (20.9) | **0.042** | 36 (31.3) | 9 (25.7) | 19 (46.3) | 8 (20.5) | **0.035** |
| **HF decompensation (n, %)** | | | | | | | | | | |
| Arrhythmia | 2 (1.7) | 1 (3.0) | 0 (0.0) | 1(2.4) | 0.744 | 2 (1.7) | 1 (2.9) | 1 (2.4) | 0 (0.0) | 0.756 |
| Infection | 40 (34.8) | 13 (39.4) | 13 (33.3) | 14 (32.6) | 0.803 | 40 (34.8) | 13 (37.1) | 14 (34.2) | 13 (33.3) | 0.937 |
| Ischemic | 0 (0.0) | 0 (0.0) | 0 (0.0) | 0 (0.0) | - | 0 (0.0) | 0 (0.0) | 0 (0.0) | 0 (0.0) | - |
| Unknown | 38 (33.0) | 11 (33.3) | 12 (30.7) | 15 (34.9) | 0.924 | 38 (33.0) | 11 (31.4) | 12 (29.3) | 15 (38.5) | 0.663 |
| **All-cause mortality (n, %)** | 32(27.8) | 8 (24.2) | 13 (33.3) | 11 (25.6) | 0.635 | 32(27.8) | 8 (22.7) | 14 (34.2) | 10 (25.6) | 0.512 |
| **Reason of death (n, %)** | | | | | | | | | | |
| HF | 10 (8.7) | 3 (9.1) | 3 (7.7) | 4 (9.3) | 1.000 | 10 (8.7) | 3 (8.6) | 3 (7.3) | 4 (10.3) | 0.919 |
| CVD | 7 (6.1) | 1 (3.0) | 5 (12.8) | 1 (2.3) | 0.148 | 7 (6.1) | 1 (2.9) | 5 (12.2) | 1 (2.6) | 0.217 |
| Other | 14 (12.2) | 3 (9.1) | 5 (12.8) | 6 (14.0) | 0.879 | 14 (12.2) | 3 (8.6) | 6 (14.6) | 5(12.8) | 0.724 |
| **Composite outcome (n, %)** | 109 (94.8) | 32 (97.0) | 38 (97.4) | 39 (90.7) | 0.476 | 109 (94.8) | 34 (97.1) | 40 (97.6) | 35(89.7) | 0.319 |
| **CVD occurrence (n, %)** | | | | | | | | | | |
| IHD | 1 (0.9) | 0 (0.0) | 0 (0.0) | 1 (2.3) | 1.000 | 1 (0.9) | 0 (0.0) | 0 (0.0) | 1 (2.6) | 0.643 |
| Stroke | 8 (7.00) | 4 (12.1) | 3 (7.7) | 1 (2.3) | 0.212 | 8 (7.00) | 4 (11.4) | 3 (7.3) | 1 (2.6) | 0.347 |
| MI | 11 (9.6) | 3 (9.1) | 3 (7.7) | 5 (11.6) | 0.922 | 11 (9.6) | 3 (8.6) | 3 (7.3) | 5 (12.8) | 0.728 |
| TIA | 6 (5.2) | 3 (9.1) | 2 (5.1) | 1 (2.3) | 0.438 | 6 (5.2) | 3 (8.6) | 2 (4.9) | 1 (2.6) | 0.513 |
| AF | 27 (23.5) | 5 (15.2) | 17 (43.6) | 5 (11.6) | <0.01 | 27 (23.5) | 5 (14.3) | 18 (43.9) | 4 (10.3) | **<0.01** |
| **Biochemical change** | | | | | | | | | | |

| | | | | | | | | | | |
|---|---|---|---|---|---|---|---|---|---|---|
| **Absolute Change of Analytical Data** | | | | | | | | | | |
| Absolute Change EF (%) (*) | 0 (-3-3) | 0(-3-1) | 0 (-5-2) | 0 (0-5) | 0.168 | 0 (-3-3) | 0(-3-1) | 0 (-5-2) | 0 (0-5) | 0.257 |
| Absolute Change LVEF (%) (*) | 0.5 (-3-6) | -2 (-6-5) | 0.5(-5.5-6) | 3 (-2-9.3) | 0.428 | 0.5 (-3-6) | -2 (-6-5) | 1(-3-6) | 3 (-3-9.3) | 0.562 |
| Absolute Change eGFR (mL/min/1.73 m²) (*) | -3(-15-7) | -3(-18-7) | -1(-10-3) | -6(-14-12) | 0.810 | -3(-15-7) | -5(-19-7) | -1(-8-3) | -4(-14-13) | 0.639 |
| Absolute Change Creatinine (mg/dL) (*) | 9 (-10-31) | 11 (-8-28) | 4 (-6-33) | 16 (-22-31) | 0.967 | 9 (-10-31) | 14 (-8-35) | 4 (-6-28) | 12 (-22-31) | 0.851 |
| Absolute Change CRP (mg/L) (*) | 1 (-25-38) | 6.5 (-20-46) | 8 (-2-65) | -18.5 (-47.5-12) | 0.114 | 1 (-25-38) | 6.5 (-20-46) | 6 (-2-46) | -25(-49-17) | 0.109 |
| Absolute Change NT-ProBNP (pg/mL) (*) | 521.5 (-178-2793) | 708.5 (0-1469) | 60 (-2773-1147) | 2194 (234-12971) | 0.068 | 521.5 (-178-2793) | 620 (-178-1469) | 60 (-1841.5-1949) | 395 0(318-13052) | **0.025** |
| Absolute Change SBP (mmHg) (*) | -6.3±29.6 | -3.1±28.4 | -4.5±31.0 | -10.4±29.4 | 0.509 | -6.3±29.6 | -5.4±31.4 | -4.9±31.3 | -8.6±26.6 | 0.838 |
| Absolute Change DBP (mmHg) (*) | -0.3±17.8 | 0.2±19.9 | 1.8±16.6 | -2.5±17.3 | 0.556 | -0.3±17.8 | -0.7±20.0 | 0.5±17.3 | -0.8±16.6 | 0.935 |
| Absolute Change Hemoglobin (g/dL) (*) | 0.1±2.0 | -0.5±2.2 | 0.5±2.1 | 0.2±1.8 | 0.113 | 0.1±2.0 | -0.4±2.1 | 0.4±2.1 | 0.3±1.8 | 0.141 |
| Absolute Change Ferritin (ug/L) (*) | 7.5 (-51-81.4) | -8.5 (-47.5-28.5) | 7 (-36.5-32.5) | 88 (-74-421) | 0.284 | 7.5 (-51-81.4) | 1 (-37-40) | 9 (-22-42) | 13 (-209-422) | 0.748 |
| Absolute Change Iron (umol/L) (*) | 1 (-1.5-5) | -0.5(-3-1) | 1 (-1-3) | 4 (2-7) | <0.01 | 1 (-1.5-5) | -1 (-3-1) | 1 (-1-3) | 4 (2-7) | **<0.01** |
| Absolute Change Transferrin (g/L) (*) | -0.1 (-0.5-0.3) | 0.2(-0.3-0.6) | -0.2 (-0.6-0.2) | -0.1 (-0.5-0.1) | 0.105 | -0.1(-0.5-0.3) | 0.1(-0.4-0.6) | -0.2 (-0.6-0.1) | -0.1 (-0.6-0.1) | 0.138 |
| Absolute Change TSAT (%) (*) | 1.9 (-1.7-11.2) | -1.5 (-6.7-1.9) | 1.4 (-1.2-4.7) | 13.1(2.4-23.2) | <0.01 | 1.9 (-1.7-11.2) | -1.6 (-6.2-1.6) | 1.6(-1.1-4.5) | 14.3(9-23.6) | **<0.01** |

Data are presented as number (percentage) for categorical variables, mean ± standard deviation (SD) for normally distributed continuous variables, and median (interquartile range [IQR]) for non-normally distributed continuous variables. Absolute changes were calculated as the follow-up value minus the baseline value. Patients with heart failure with preserved ejection fraction (HFpEF) were classified as having untreated iron deficiency (ID), treated ID, or no ID according to the American Heart Association (AHA) and European Society of Cardiology (ESC) definitions. P-values represent comparisons among the three groups within each definition. Variables marked with an asterisk (*) were analyzed as continuous variables.

**Supplementary Table S9.** Comparison of Clinical Adverse Outcomes Between Iron Deficiency (ID) and Non-Iron Deficiency (Non-ID) Groups in Patients with Heart Failure with Preserved Ejection Fraction (HFpEF)

| Category | AHA-HF ID definition | | | | ESC-HF ID definition | | | |
|---|---|---|---|---|---|---|---|---|
| | Total number | ID | Not ID | p-value | Total number | ID | Not ID | p-value |
| | n=115 | n=72 | n=43 | | n=115 | n=76 | n=39 | |
| **Clinical adverse outcome** | | | | | | | | |
| **Emergency visits (n, %)** | | | | | | | | |
| Total Emg | 107 (93.0) | 69 (95.8) | 38 (88.4) | 0.148 | 107 (93.0) | 73 (96.1) | 34 (87.2) | 0.118 |
| Total number of Emg (*) | 3 (2-6) | 3 (2-5.5) | 3 (1-6) | 0.993 | 3 (2-6) | 3 (2-5.5) | 3 (1-6) | 0.774 |
| HF-Emg | 78 (67.8) | 53 (73.6) | 25 (58.1) | 0.086 | 78 (67.8) | 55 (72.4) | 23 (59.0) | 0.145 |
| CVD-emg | 28 (24.4) | 20 (27.8) | 8 (18.6) | 0.267 | 28 (24.4) | 21 (27.6) | 7 (18.0) | 0.252 |
| **Admission (n, %)** | | | | | | | | |
| Total- admission | 104 (90.4) | 69 (95.8) | 35 (81.4) | 0.019 | 104 (90.4) | 72 (94.7) | 32 (82.1) | 0.043 |
| Length of hospital stay (days) (*) | 2 (1-4) | 2 (1-3.5) | 2 (1-4) | 0.715 | 2 (1-4) | 2 (1-3.5) | 2 (1-4) | 0.768 |
| HF-admission | 72 (62.6) | 48 (66.7) | 24 (55.8) | 0.244 | 72 (62.6) | 50 (65.8) | 22 (56.4) | 0.325 |
| CVD- admission | 36 (31.3) | 27 (37.5) | 9 (20.9) | 0.064 | 36 (31.3) | 28 (36.8) | 8 (20.5) | 0.074 |
| **HF decompensation (n, %)** | | | | | | | | |
| Arrhythmia | 2 (1.7) | 1 (1.4) | 1 (2.3) | 1.000 | 2 (1.7) | 2 (2.6) | 0 (0.0) | 0.548 |
| Infection | 40 (34.8) | 26 (36.1) | 14 (32.6) | 0.699 | 40 (34.8) | 27 (35.5) | 13 (33.3) | 0.815 |
| Ischemic | 0 (0.0) | 0 (0.0) | 0 (0.0) | - | 0 (0.0) | 0 (0.0) | 0 (0.0) | - |
| Unknown | 38 (33.0) | 23 (31.9) | 15 (34.9) | 0.746 | 38 (33.0) | 23 (30.3) | 15 (38.5) | 0.376 |
| **All-cause mortality (n, %)** | 32 (27.8) | 21 (29.2) | 11 (25.6) | 0.678 | 32 (27.8) | 22 (29.0) | 10 (25.6) | 0.708 |
| **Reason of death (n, %)** | | | | | | | | |
| HF | 10 (8.7) | 6 (8.3) | 4 (9.3) | 1.000 | 10 (8.7) | 6 (7.9) | 4 (10.3) | 0.732 |
| CVD | 7 (6.1) | 6 (8.3) | 1 (2.3) | 0.254 | 7 (6.1) | 6 (7.9) | 1 (2.6) | 0.420 |
| Other | 14 (12.2) | 8 (11.1) | 6 (14.0) | 0.652 | 14 (12.2) | 9 (11.8) | 5 (12.8) | 0.879 |
| **Composite outcome (n, %)** | 109 (94.8) | 70 (97.2) | 39 (90.7) | 0.194 | 109 (94.8) | 74 (97.4) | 35 (89.7) | 0.178 |
| **CVD occurrence (n, %)** | | | | | | | | |
| IHD | 1 (0.9) | 0 (0.0) | 1 (2.3) | 0.374 | 1 (0.9) | 0 (0.0) | 1 (2.6) | 0.339 |
| Stroke | 8 (7.00) | 7 (9.7) | 1 (2.3) | 0.255 | 8 (7.00) | 7 (9.2) | 1 (2.6) | 0.262 |
| MI | 11 (9.6) | 6 (8.3) | 5 (11.6) | 0.561 | 11 (9.6) | 6 (7.9) | 5 (12.8) | 0.395 |
| TIA | 6 (5.2) | 5 (6.9) | 1 (2.3) | 0.408 | 6 (5.2) | 5 (6.6) | 1 (2.6) | 0.662 |
| AF | 27 (23.5) | 22 (30.6) | 5 (11.6) | 0.021 | 27 (23.5) | 23 (30.3) | 4 (10.3) | **0.020** |
| **Biochemical change** | | | | | | | | |
| **Absolute Change of Analytical Data** | | | | | | | | |
| Absolute Change EF (%) (*) | 0 (-3-3) | 0 (-5-1.5) | 0 (0-5) | 0.060 | 0 (-3-3) | 0 (-4.5-1.5) | 0 (0-5) | 0.099 |
| Absolute Change LVEF (%) (*) | 0.5 (-3-6) | -1 (-6-5) | 3 (-2-9.3) | 0.230 | 0.5 (-3-6) | 0 (-6-6) | 2 (-3-9.3) | 0.410 |

| | | | | | | | | |
|---|---|---|---|---|---|---|---|---|
| Absolute Change eGFR (mL/min/1.73 m²) (*) | -3 (-15-7) | -1 (-15-5) | -6 (-14-12) | 0.882 | -3 (-15-7) | -1.5 (-15-5) | -4 (-14-13) | 0.720 |
| Absolute Change Creatinine (mg/dL) (*) | 9 (-10-31) | 6 (-7-30.5) | 16 (-22-31) | 0.993 | 9 (-10-31) | 7.5 (-7-30.5) | 12 (-22-31) | 0.820 |
| Absolute Change CRP (mg/L) (*) | 1 (-25-38) | 8 (-4-50) | -18.5 (-47-12) | 0.042 | 1 (-25-38) | 6 (-4-50) | -25 (-49-17) | **0.039** |
| Absolute Change NT-ProBNP (pg/mL) (*) | 521.5 (-178-2793) | 255 (-981-1449.5) | 2194 (234-12971) | 0.037 | 521.5 (-178-2793) | 220 (-910-1469) | 3950 (318-13052) | **<0.01** |
| Absolute Change SBP (mmHg) (*) | -6.3±29.6 | -3.9±29.7 | -10.4±29.4 | 0.251 | -6.3±29.6 | -5.1±31.1 | -8.6±26.6 | 0.555 |
| Absolute Change DBP (mmHg) (*) | -0.3±17.8 | 1.0±18.1 | -2.4±17.3 | 0.309 | -0.3±1.7 | -0.2±18.5 | -0.8±16.6 | 0.831 |
| Absolute Change Hemoglobin (g/dL) (*) | 0.1±2.0 | 0.1±2.2 | 0.2±1.8 | 0.663 | 0.1±2.0 | 0.02±2.1 | 0.3±1.8 | 0.421 |
| Absolute Change Ferritin (ug/L) (*) | 7.5 (-51-81.4) | 0.5 (-44-28.5) | 88 (-74-421) | 0.122 | 7.5 (-51-81.4) | 6 (-37-42) | 13 (-209-422) | 0.465 |
| Absolute Change Iron (umol/L) (*) | 1 (-1.5-5) | 0 (-2-2) | 4 (2-7) | <0.01 | 1 (-1.5-5) | 0 (-2-2) | 4 (2-7) | **<0.01** |
| Absolute Change Transferrin (g/L) (*) | -0.1(-0.5-0.3) | -0.1(-0.5-0.4) | -0.1 (-0.5-0.1) | 0.452 | -0.1(-0.5-0.3) | -0.1(-0.5-0.4) | -0.1 (-0.6-0.1) | 0.458 |
| Absolute Change TSAT (%) (*) | 1.9 (-1.7-11.2) | 0.2 (-2.6-4) | 13.1(2.4-23.2) | <0.01 | 1.9 (-1.7-11.2) | 0.2 (-2.8-4) | 14.3(9-23.6) | **<0.01** |

Data are presented as number (percentage) for categorical variables, mean ± standard deviation (SD) for normally distributed continuous variables, and median (interquartile range [IQR]) for non-normally distributed continuous variables. Absolute changes were calculated as the follow-up value minus the baseline value. Patients with heart failure with preserved ejection fraction (HFpEF) were classified as having iron deficiency (ID) or no ID according to the American Heart Association (AHA) and European Society of Cardiology (ESC) definitions. P-values represent comparisons between the ID and non-ID groups within each definition. Variables marked with an asterisk (*) were analyzed as continuous variables.

**Supplementary Table S10** Multivariable logistic regression analysis for predictors of ID , hospital admission, EMG and Composite outcome

| Predictor | Odds Ratio (OR) | 95% CI | P-value |
|---|---|---|---|
| **Predictors of Iron deficiency** | | | |
| **BMI** | 1.07 | 1.02-1.13 | **<0.01** |
| **Age** | 1.01 | 0.99,1.04 | 0.376 |
| **Female** | 1.64 | 0.89,3.07 | 0.114 |
| **DM** | 1.65 | 0.84,3.24 | 0.114 |
| **hypertension** | 1.56 | 0.69,3.46 | 0.278 |
| **Anemia** | 0.98 | 0.28,3.4 | 0.970 |
| **TI/stroke** | 0.58 | 0.10-3.28 | 0.536 |
| **Predictors of admission** | | | |
| **Iron profile** | | | |
| **Transferrin** | 0.246 | 0.09-0.70 | **<0.01** |
| **TSAT** | 0.858 | 0.79-0.93 | **<0.01** |
| **Predictors of EMG** | | | |
| **Iron profile** | | | |
| **Transferrin** | 0.057 | 0.01-0.35 | **<0.01** |
| **TSAT** | 0.827 | 0.73-0.93 | **<0.01** |
| **Predictors of Composite outcome** | | | |
| **Iron profile** | | | |
| **Transferrin** | 0.161 | 0.02-09.7 | **0.046** |
| **Iron** | 0.738 | 0.59-0.92 | **<0.01** |

Results are presented as adjusted odds ratios (ORs) with 95% confidence intervals (CIs) and corresponding P-values. BMI, body mass index; DM, diabetes mellitus; TIA, transient ischemic attack; TSAT, transferrin saturation; EMG, emergency department visit. represent comparisons of absolute changes among the three corresponding groups. Variables marked with an asterisk (*) were analyzed as continuous variables.

## Supplementary Table S11. Covariate balance before and after entropy balancing

| Variable | Before entropy balancing | | | After entropy balancing (ATT) | | |
|---|---|---|---|---|---|---|
| | **Supplemented (n=67)** | **Non-supplemented (n=62)** | **SMD** | **Supplemented (n=60)** | **Non-supplemented (weighted controls; 47 used)** | **SMD** |
| BMI (kg/m²) | 28.93 | 28.08 | 0.127 | 28.91 | 28.83 | 0.013 |
| History of TIA/stroke, % | 1.5 | 17.7 | 0.569 | 1.7 | 1.3 | 0.013 |
| HF type 2, % | 9.0 | 16.1 | 0.216 | 10.0 | 11.4 | 0.041 |
| HF type 3, % | 65.7 | 48.4 | 0.352 | 65.0 | 61.5 | 0.072 |
| EF (%) | 48.5 | 45.6 | 0.195 | 48.9 | 47.9 | 0.066 |
| LVEF (%) | 50.5 | 46.2 | 0.294 | 50.4 | 49.1 | 0.087 |
| Hydralazine use, % | 37.3 | 22.6 | 0.323 | 35.0 | 33.8 | 0.027 |
| Hemoglobin | 8.88 | 10.03 | 0.686 | 9.05 | 9.25 | 0.118 |
| Ferritin | 175.4 | 272.5 | 0.341 | 173.7 | 174.0 | 0.001 |
| Serum iron | 7.28 | 7.74 | 0.057 | 6.27 | 6.50 | 0.029 |
| Transferrin | 2.30 | 2.11 | 0.281 | 2.24 | 2.25 | 0.018 |
| TSAT (%) | 11.3 | 15.9 | 0.609 | 11.9 | 12.2 | 0.047 |

Values are means for continuous variables and percentages for binary variables. SMDs are shown as absolute standardized mean differences. After entropy balancing, all covariates had an SMD below 0.10 except hemoglobin (SMD=0.118), indicating good overall balance with minor residual imbalance in hemoglobin. ATT, average treatment effect on the treated; EF, ejection fraction; HF, heart failure; LVEF, left ventricular ejection fraction; TIA, transient ischemic attack; TSAT, transferrin saturation.
